# A Conserved DT2‐bZIP66‐NF‐YC4 Regulatory Module Confers Drought Tolerance in Rice and *Arabidopsis*

**DOI:** 10.1002/advs.76034

**Published:** 2026-06-09

**Authors:** Jun Shen, Yuchen Li, Liang Zhang, Xiaosong Ma, Chenyin Peng, Chunting Yuan, Yu Wu, Kaiyu Zhao, Sheng Zhao, Jiazhuo Guo, Run Li, Ziqian Yan, Shule Ren, Yige Han, Xin Lin, Xinyue Li, Ming Li, Pingfang Yang, Shiyong Song

**Affiliations:** ^1^ State Key Laboratory of Rice Biology and Breeding, Zhejiang Provincial Key Laboratory of Crop Genetic Resources, College of Agriculture and Biotechnology Zhejiang University Hangzhou China; ^2^ College of Agronomy, Bio‐breeding Laboratory of Anhui Province Anhui Agricultural University Hefei China; ^3^ Shanghai Agrobiological Gene Center Shanghai China; ^4^ State Key Laboratory of Biocatalysis and Enzyme Engineering, School of Life Sciences Hubei University Wuhan China

**Keywords:** drought tolerance, DT2‐bZIP66‐NF‐YC4, OsDT2, rice and *Arabidopsis*

## Abstract

Drought stress severely restricts plant growth and crop productivity. Although numerous transcription factors have been linked to drought responses, the regulatory networks underlying drought tolerance in rice remain incompletely understood. Here, through a CRISPR/Cas9‐based genetic screen of a rice mutant population, we identified a mutant with decreased drought tolerance, *osdt2*. *OsDT2* encodes a CCCH‐type zinc‐finger protein and its overexpression confers strong drought resistance. We demonstrate that OsDT2 physically interacts with the bZIP transcription factor OsbZIP66, and they synergistically activate drought‐responsive genes, such as *LATE EMBRYOGENESIS ABUNDANT 3* (*OsLEA3*), to enhance drought tolerance. Furthermore, the nuclear factor Y subunit C4 (OsNF‐YC4) interacts with both OsDT2 and OsbZIP66, coordinating their recruitment to the promoter of *OsLEA3*. Remarkably, the OsDT2 function is conserved across cereals, including wheat and maize, and the tripartite DT2‐bZIP66‐NF‐YC4 module is functionally conserved in mediating drought responses in both rice and *Arabidopsis*. Collectively, our study reveals a novel, evolutionarily conserved transcriptional regulatory module that fine‐tunes drought‐responsive gene expression and provides potential targets for engineering drought‐resilient crops.

## Graphical abstract

This study identifies a conserved tripartiteDT2–bZIP66–NF‐YC4 transcriptional module that enhances rice drought tolerance by activating stress‐responsive genes. This regulatory complex is functionally conserved across cereals and Arabidopsis, providing promising targets for engineering drought‐resilient crops.

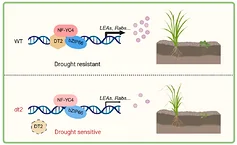

## 1 Introduction

Drought is a major environmental constraint on plant growth and agricultural productivity. Sessile plants have evolved a range of adaptive strategies to perceive and respond to drought stress, including morphological and structural adjustments, activation of drought‐responsive genes, hormone production, and the accumulation of osmotic regulators [1, 2, 3]. During this process, numerous drought‐responsive genes are induced, and transcription factors play central roles in the accompanying transcriptional reprogramming, including members of the AP2/ERF, MYB, bZIP, NAC and zinc‐finger families [4, 5, 6, 7].

Among these regulators, CCCH zinc‐finger proteins are characterized by a zinc‐binding motif containing three cysteines and one histidine and are broadly conserved across eukaryotes. Genome‐wide surveys identified 68 CCCH genes in Arabidopsis and 67 in rice [8], and functional studies have implicated this family in plant development as well as in responses to abiotic and biotic stresses [9, 10, 11, 12, 13, 14]. In *Arabidopsis*, overexpression of *AtTZF1* enhances drought resistance, whereas *AtTZF2‐* and *AtTZF3‐* overexpressing lines exhibit heightened ABA sensitivity and increased drought tolerance [15, 16]. In rice, several CCCH genes, including *OsTZF1*, *OsTZF5*, *OsTZF7*, *OsC3H10*, and *OsC3H47* are inducible by abscisic acid and drought conditions; their overexpression markedly improves drought tolerance in seedlings [17, 18, 19, 20, 21]. Furthermore, OsC3H positively regulates *Osr40C1* expression to promote drought tolerance and the expression of *Osr40C1* is positively correlated with drought resistance in *indica* rice cultivars [22]. Collectively, these studies support CCCH proteins as important drought regulators; nevertheless, the molecular mechanisms underlying the drought response of most zinc finger proteins in rice remain largely elusive.

The basic leucine zipper (bZIP) factors constitute another major regulatory layer in drought signaling. In *Arabidopsis*, 75 putative bZIP members have been identified [23], several of which function in water‐deficit responses, including ABSCISIC ACID RESPONSIVE ELEMENTS‐BINDING PROTEIN 2 (AREB1), AREB2, ABSCISIC ACID RESPONSIVE ELEMENTS‐BINDING FACTOR 3 (ABF3), bZIP62, and TGACG MOTIF‐BINDING FACTOR 4 (TGA4) [24, 25, 26]. Rice contains 89 predicted bZIP genes [27], and multiple members, including TRAB1/OsbZIP66, OsbZIP23, OsbZIP46, OsbZIP72, and OsbZIP71, positively regulate drought tolerance [4, 28, 29, 30, 31, 32, 33]. However, how rice bZIP factors coordinate with other transcription regulators to activate key drought‐responsive genes remains largely unclear.

NUCLEAR FACTOR‐Y (NF‐Y) is a conserved heterotrimeric complex composed of NF‐YA, NF‐YB and NF‐YC subunits that binds CCAAT‐containing cis‐elements to regulate target‐gene transcription [34, 35]. NF‐YC, also termed HISTONE‐ASSOCIATED PROTEIN5 (HAP5) or CCAAT BINDING FACTOR C (CBF‐C) [36], functions as an important participant in various developmental and stress responses. In *Arabidopsis*, NF‐YC3, NF‐YC4, and NF‐YC9 function redundantly to modulate seed germination mediated by GA and ABA, as well as the light‐inhibited hypocotyl elongation pathway [37, 38]. Moreover, CONSTANS (CO) protein forms co‐phase condensates with NF‐YB2/NF‐YC9/*Flowering Locus T* (*FT*) to precisely control heterogeneous CO assembly and *FT* transcriptional activation [39]. In rice, the gene QT12 is controlled by upstream NF‐Y transcription factors, contributing to the balance of storage substances [40]. In soybean, GmNF‐YC14 forms a heterotrimer with GmNF‐YA16 /GmNF‐YB2 to activate the ABA signaling pathway and enhance stress tolerance [41]. These studies suggest that NF‐YCs participate in hormone and stress pathways, yet whether rice NF‐YCs act as assembly factors coordinating transcription factors on drought‐inducible promoters remains unclear.

Here, we performed a forward genetic screen for altered drought responses in rice and named them as *drought tolerance x [dt(x)]*. We identified OsDT2, a CCCH zinc‐finger protein, as a previously uncharacterized positive regulator of drought tolerance. We show that OsDT2 cooperates with OsbZIP66 and OsNF‐YC4 to activate *OsLEA3* expression, thereby promoting drought resistance. We further provide evidence that this transcriptional regulatory module is conserved in rice and *Arabidopsis*. These findings reveal a conserved mechanism by which a CCCH zinc‐finger protein coordinates with bZIP and NF‐Y factors to fine‐tune drought‐inducible gene expression in plants.

## 2 Results

### 2.1 *osdt2* is Sensitive to Drought

**FIGURE 1:**
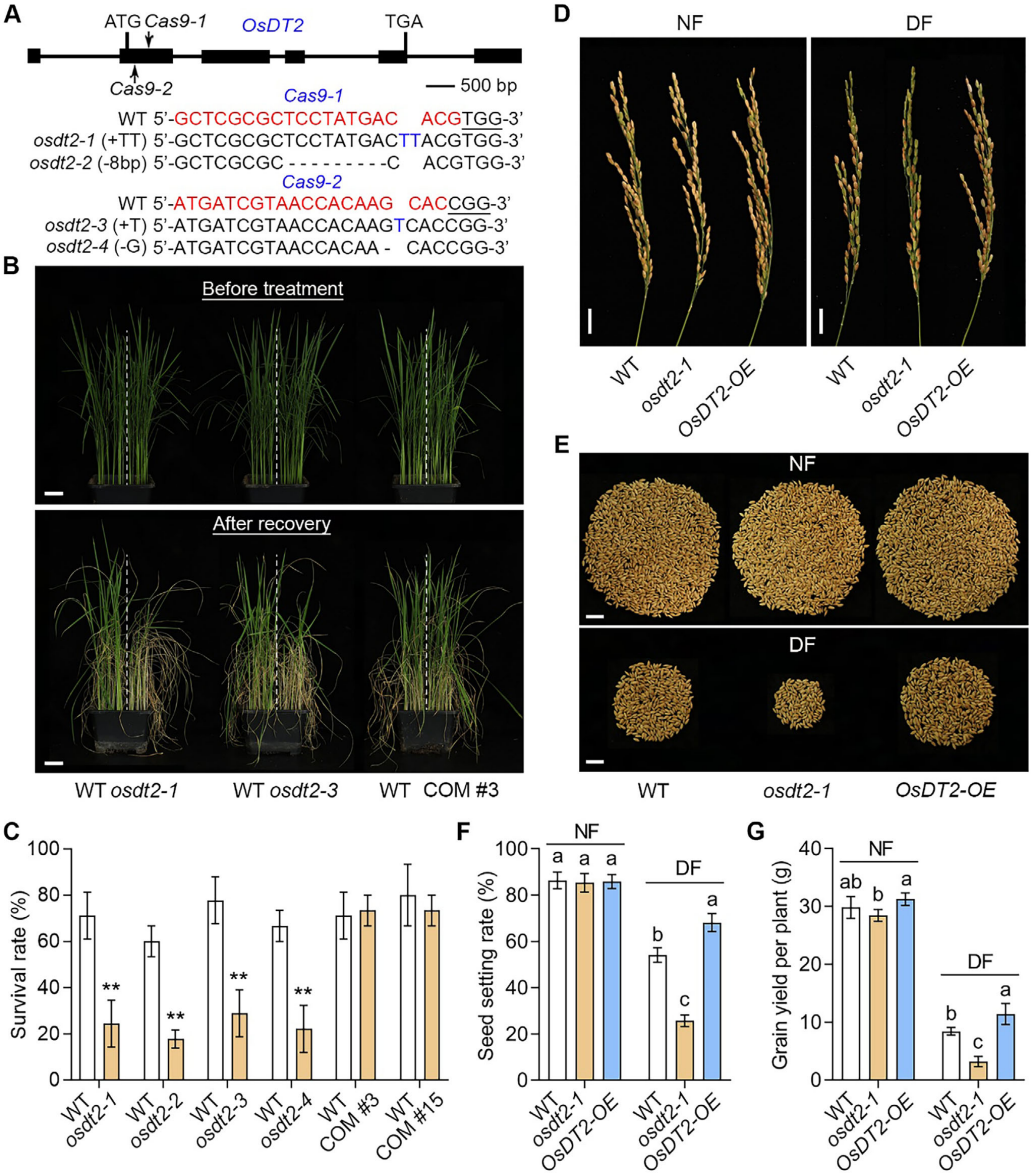
*OsDT2* positively regulates drought response in rice. (A) CRISPR/Cas9‐mediated target mutagenesis of *OsDT2*. The schematic diagram shows the *OsDT2* gene harboring the CRISPR/Cas9 target sites indicated by arrows. Exons and introns are represented by black boxes and lines, respectively. Alignments between wild‐type and mutated sequences containing the target sites are shown below the schematic diagrams. (B) Plant morphologies of wild type, *osdt2*, and COM #3 (the complementation line, *osdt2‐1 gOsDT2‐3FLAG* #3) before drought treatment and after recovery. Scale bars, 5 cm. (C) Survival rates of various genotypes after recovery from water deprivation. Values are mean ± SD (*n* = 3 biological replicates). Asterisks indicate significant differences between wild‐type and other plants (two‐tailed Student's *t*‐test, **, *p* < 0.01). (D and E) Images of representative panicles and grain yield per plant of *osdt2‐1*, and *OsDT2‐OE* #28 (*Ubi:OsDT2‐3FLAG*) plants under normal field (paddy field) (NF) or drought field (DF). Scale bars, 2 cm. (F and G) Seed‐setting rate and grain yield per plant of *osdt2‐1* and *OsDT2‐OE* #28 under NF and DF, respectively. Data are the mean ± SD (*n* = 10 samples for each genotype). Different letters indicate notable differences determined by one‐way ANOVA followed by Tukey's test (*p* < 0.05).

A forward screen of a genome‐scale CRISPR/Cas9‐mutagenized rice population in the ZH11 background identified a drought‐sensitive mutant, *osdt2* (Figure S1A). When seedlings were grown in split pots and subjected to water withdrawal, *osdt2* showed a dramatically lower survival rate after re‐watering in comparison with ZH11 (Figure S1B,C). Sequencing identified a 1‐bp guanine (G) insertion in the second exon of *OsDT2* (LOC_Os04g32340), resulting in a premature stop codon (Figure S1D,E). To further validate gene function, we designed two single guide RNAs (sgRNAs) targeting the *OsDT2* exon in the *Nipponbare* background using the CRISPR/Cas9 system. The resulting homozygous loss‐of‐function mutants, which no longer carried the CRISPR/Cas9 vector, were named *osdt2‐1* to *osdt2‐4* (Figure 1A). Drought‐tolerance assays showed that *osdt2* mutants displayed a markedly more sensitive phenotype than the wild type under water‐deficit conditions, together with a significantly reduced survival rate after rewatering (Figure 1B,C). To confirm that the stress‐sensitive phenotype of *osdt2* was caused by loss of *OsDT2* function, we introduced genomic *gOsDT2* or *gOsDT2‐3FLAG* constructs into homozygous *osdt2‐1* calli. This construct contained an 8.62‐kb genomic fragment comprising a 2.0‐kb upstream promoter region and a 6.62‐kb genomic coding region with introns, fused in frame to a 3FLAG tag. More than 23 independent transformants carrying a single‐copy transgene were identified based on a 3:1 Mendelian segregation ratio. To verify the effectiveness of the complementation, the expression levels of *OsDT2* in the complementation lines were examined, confirming successful restoration of *OsDT2* expression (Figure S1F). Phenotypic analysis further showed that all T2 transgenic lines displayed partial to substantial rescue of the drought‐sensitive phenotype of *osdt2* (Figure 1B,C), indicating that *OsDT2* is responsible for the drought‐sensitive phenotype of *osdt2*. Among them, *osdt2‐1 gOsDT2‐3FLAG* #3, which showed drought sensitivity comparable to that of wild‐type seedlings, was selected for subsequent investigation. We also generated *OsDT2* overexpression lines. Among the 20 lines obtained, 4 lines were randomly chosen for detailed analysis. In these lines, *OsDT2* transcript levels were markedly increased, and all exhibited enhanced drought tolerance relative to the wild type, with the magnitude of the tolerant phenotype closely correlating with the level of transgene expression (Figure S1G–I). Together, these results identify *OsDT2* as a positive regulator of drought tolerance.

Furthermore, we conducted a comprehensive yield‐trait evaluation throughout the entire growth cycle of rice under normal and aerobic drought field conditions. Under normal conditions, the *osdt2‐1* and *OsDT2‐OE* plants exhibited comparable seed‐setting rates and grain yield per plant (Figure 1E–G). Furthermore, no significant differences among the wild type, *osdt2‐1*, and *OsDT2‐OE* plants were observed for agronomic traits, including panicle length, grain length, grain width, 1000‐grain weight and tiller number (Figure S2). During the stress treatment, *osdt2‐1* displayed a significantly lower seed‐setting rate and grain yield per plant, while *OsDT2‐OE* plants showed a marked increase in both traits compared with the wild type (Figure 1D–G and Figure S1J). Collectively, these results demonstrate that OsDT2 regulates drought resistance, holding great potential in drought resistance breeding in rice.

### 2.2 OsDT2 Interacts with OsbZIP66

To investigate the function of OsDT2, we first examined its expression patterns across different tissues of wild‐type plants. *OsDT2* was expressed in all tissues tested, with the highest transcript levels observed in leaves (Figure S3A). Its expression was also significantly upregulated following ABA and PEG treatments (Figure S3B). Histochemical staining of transgenic leaves carrying a *ProOsDT2:GUS* reporter construct showed that GUS activity was enhanced by both ABA and drought treatment (Figure S3C,D). In addition to ABA and PEG treatments, *OsDT2* expression was also strongly induced under drought conditions, showing a dynamic increase during stress and a decrease upon recovery (Figure S3E). Furthermore, domain analysis indicated that OsDT2 contains a CCCH‐type zinc finger domain and an RNA‐recognition motif. Consistent with this nuclear protein signature, transient expression of OsDT2‐GFP in rice protoplasts confirmed its exclusive localization in the nucleus (Figure S3F). In addition to drought stress, *OsDT2* expression responded to various other abiotic stresses and hormone treatments (Figure S3G–N).

**FIGURE 2:**
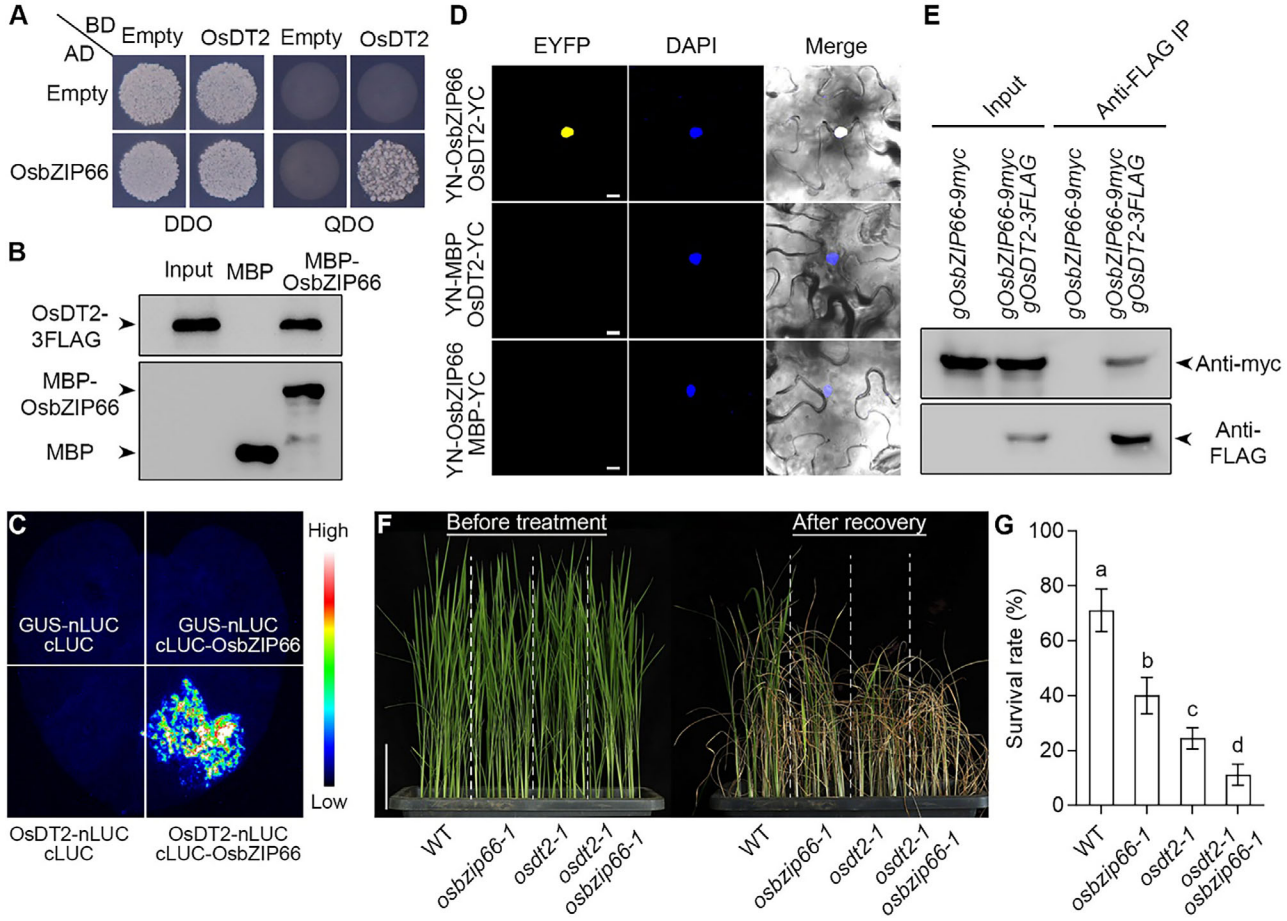
OsDT2 interacts with OsbZIP66. (A) Yeast two‐hybrid (Y2H) assay of interaction between OsDT2 and OsbZIP66. Transformed yeast cells were grown on DDO (left panel) and QDO (right panel) medium. Empty denotes AD‐ or BD‐containing vector only. (B) MBP pull‐down assay of the interaction between OsDT2 and OsbZIP66. OsDT2‐3FLAG protein extracted from *Ubi:OsDT2‐3FLAG* #28 transgenic plant was incubated with immobilized MBP or MBP‐OsbZIP66, respectively. Immunoblot analysis was performed using anti‐FLAG (upper) or anti‐MBP (lower) antibody. Input, 5% of the protein extracted from the transgenic plant. (C) LCI assay of interaction between OsDT2 and OsbZIP66. The indicated fusion pairs were coexpressed in *N. benthamiana* leaves. GUS‐nLUC and cLUC were used as negative controls. (D) BiFC analysis showing the interaction of OsDT2 and OsbZIP66. Merge, merge of EYFP, DAPI and bright field images. Scale bars, 10 µm. (E) CoIP assay demonstrating the in vivo interaction of OsDT2 and OsbZIP66 in rice leaves. Total extracts from leaves of *gOsbZIP66‐9myc* and *gOsbZIP66‐9myc gOsDT2‐3FLAG* plants were immunoprecipitated by anti‐FLAG magnetic beads. The input and immunoprecipitated protein were detected by either anti‐myc (upper panel) or anti‐FLAG (lower panel) antibody. (F and G) Representative images and survival rates of various genotypes after recovery from water deprivation. Values are mean ± SD (*n* = 3 biological replicates). One‐way ANOVA followed by Tukey's test (*p* < 0.05) was used for statistical analysis.

To identify potential components involved in the OsDT2‐mediated drought response, we conducted a yeast two‐hybrid screening using OsDT2 as a bait. Among the interactors identified was the rice basic leucine zipper (bZIP) transcription factor OsbZIP66, which has been implicated in stress responses in previous studies [4, 32, 42]. The interaction between OsDT2 and OsbZIP66 was confirmed through multiple complementary approaches. First, yeast two‐hybrid assays showed a strong interaction between OsDT2 and OsbZIP66 (Figure 2A). Pull‐down analysis further demonstrated that OsDT2 could be precipitated by MBP‐OsbZIP66 but not by MBP alone (Figure 2B). In addition, luciferase complementation imaging (LCI) confirmed their interaction in plant cells (Figure 2C). Bimolecular fluorescence complementation (BiFC) assays revealed that co‐expression of nEYFP‐OsbZIP66 and OsDT2‐cEYFP reconstituted YFP fluorescence in the nucleus, while control combinations did not (Figure 2D). Finally, to validate the interaction in rice, we crossed the functional complementation line *osbzip66‐1 gOsbZIP66‐9myc* (#13)—which fully rescues the drought‐sensitive phenotype of *osbzip66‐1* [4]—with *osdt2‐1 gOsDT2‐3FLAG #3* to obtain *gOsbZIP66‐9myc gOsDT2‐3FLAG* plants. Co‐immunoprecipitation (CoIP) assays using these plants confirmed the in vivo association between OsDT2 and OsbZIP66 (Figure 2E). Taken together, these results substantiate that OsDT2 interacts with OsbZIP66 both in vitro and in vivo.

We next explored the biological relevance of the OsDT2‐OsbZIP66 interaction through genetic analysis. Given that both OsDT2 and OsbZIP66 positively regulate drought tolerance, we compared the survival rates under drought stress among relevant genotypes. The *osdt2‐1* and *osbzip66‐1* single mutants each exhibited significantly reduced survival compared with the wild type, while the double mutant showed an even stronger drought‐sensitive phenotype, indicating that OsDT2 and OsbZIP66 cooperatively contribute to drought tolerance (Figure 2F,G). Together with the physical interaction demonstrated earlier, these results support a model in which OsDT2 and OsbZIP66 act cooperatively to regulate drought tolerance in rice.

### 2.3 OsDT2 and OsbZIP66 Share a Common Set of Target Genes

**FIGURE 3:**
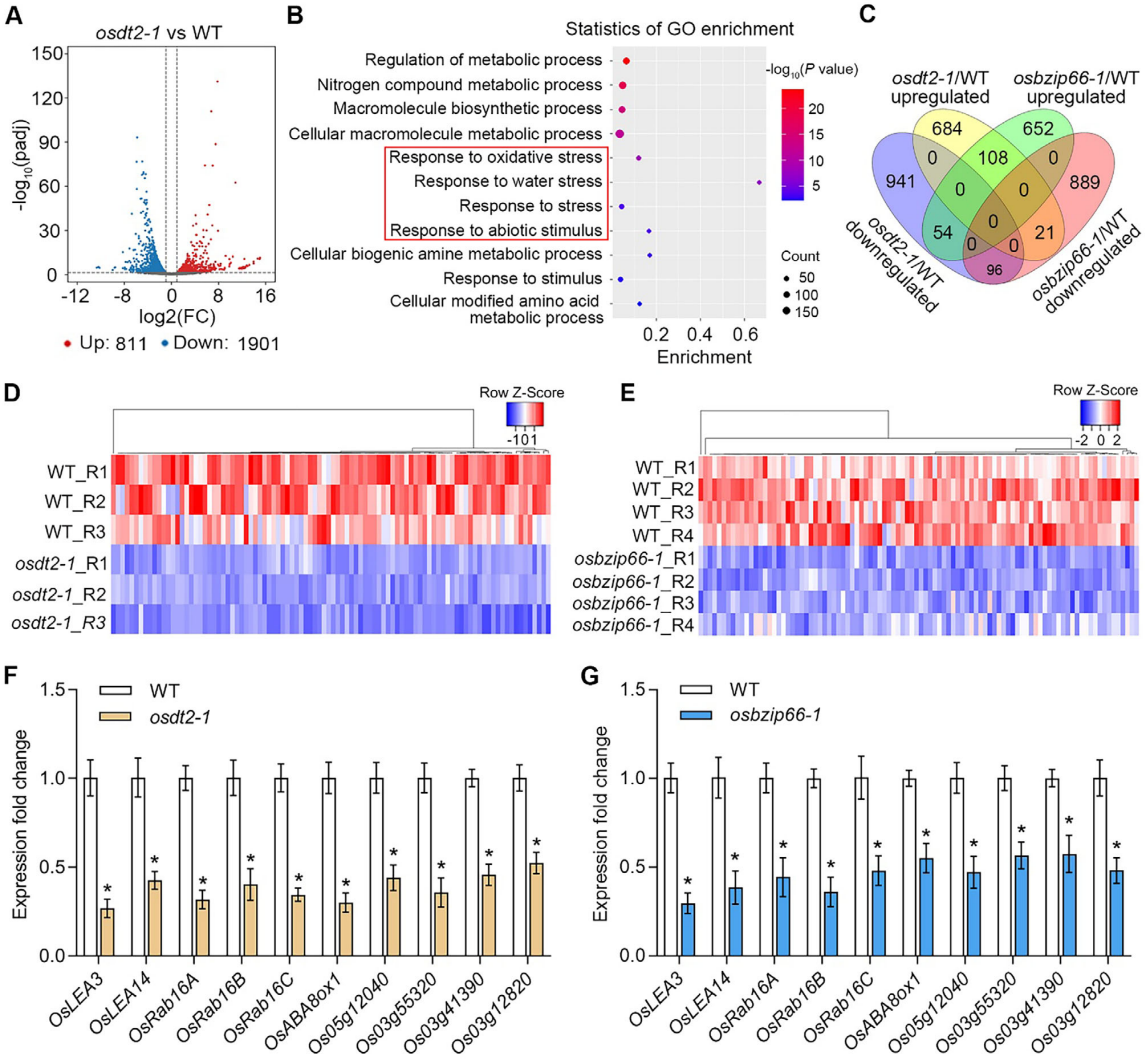
Genome‐wide transcriptional profiles of OsDT2 and OsbZIP66. (A) Volcano plots illustrating the genome‐wide expression profiles in *osdt2‐1* compared with wild‐type plants after water deprivation. Four‐week‐old wild‐type and *osdt2‐1* seedlings were exposed to 7‐d drought treatment and collected for RNA‐seq analysis. (B) Gene ontology enrichment analyses of genes down‐regulated in *osdt2‐1* after drought treatment. (C) Venn diagram of differentially expressed genes (DEGs) in *osdt2‐1* and *osbzip66‐1* mutants compared with wild type. (D and E) Expression heatmap of 96 downregulated genes selected from overlapped DEGs of two RNA‐seq results. (F and G) Quantitative real‐time PCR (qRT‐PCR) validation of the expression levels of 10 stress‐related genes in the *osdt2‐1* and *osbzip66‐1* mutants. Data represent mean ± SD (*n* = 3 biological replicates). Asterisks denote significant differences between wild type and *osdt2‐1* or *osbzip66‐1* (two‐tailed Student's *t*‐test, *, *p* < 0.05).

Based on the observed interaction between OsDT2 and OsbZIP66 and their shared drought‐sensitive mutant phenotypes, we next investigated the underlying mechanism of their cooperation in drought response. Transcriptome analysis of leaves collected from wild‐type and *osdt2‐1* plants after 7 d of drought treatment identified 811 up‐regulated and 1901 down‐regulated differentially expressed genes (DEGs) in the mutant (Figure 3A and Figure S4A). Gene ontology enrichment highlighted stress‐responsive pathways among the DEGs (Figure 3B), consistent with a positive role for OsDT2 in drought tolerance. Integrating these data with previously published transcriptomic results for *osbzip66‐1* [4] revealed 279 genes co‐regulated by both mutants. Given that OsDT2 and OsbZIP66 both act as positive regulators of drought tolerance (Figures 1, 2G and Figure S1), we focused on the 96 genes down‐regulated in both *osdt2‐1* and *osbzip66‐1* (Figure 3C–E). Among these, ten were selected for further study based on known or predicted roles in stress response, including genes encoding LEA proteins, dehydrins, ABA 8’‐hydroxylase, and several transcription factors (Figure 3D,E). Quantitative real‐time PCR (qRT‐PCR) confirmed that all ten genes were down‐regulated in both mutants (Figure 3F,G) and up‐regulated in *OsDT2‐OE* and *OsbZIP66‐OE* lines [4] (Figure S4B,C). Furthermore, ChIP‐qPCR results demonstrated that OsDT2 and OsbZIP66 directly bound to the promoters of these ten genes (Figure S5). Together, these findings reveal a coregulated gene network through which OsDT2 and OsbZIP66 jointly enhance drought tolerance, providing a mechanistic basis for their cooperative function in the drought‐responsive pathway.

### 2.4 OsDT2 and OsbZIP66 Synergistically Promote The Expression of *OsLEA3*

**FIGURE 4:**
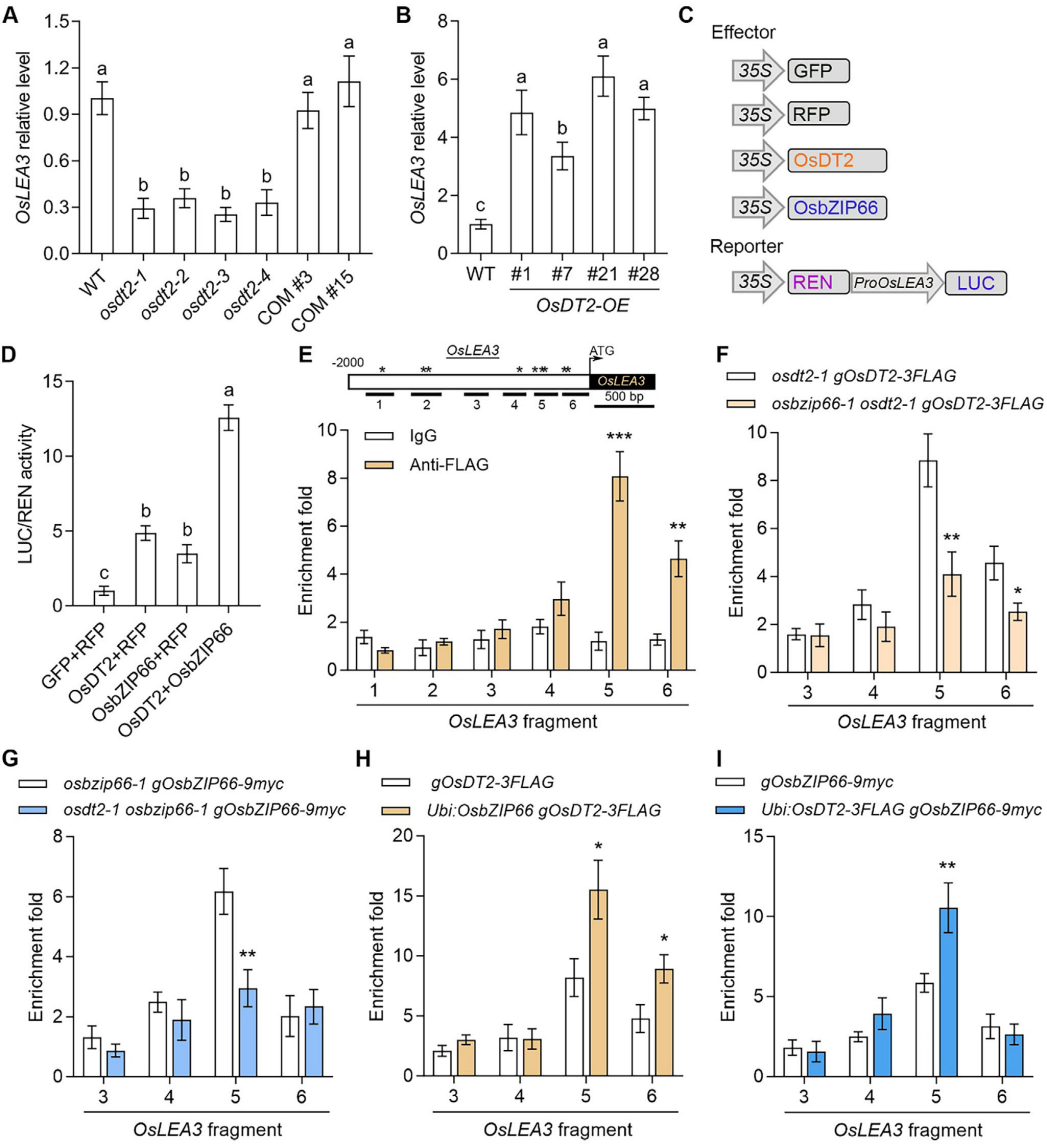
OsDT2 and OsbZIP66 synergistically regulate the expression of *OsLEA3*. (A and B) qRT‐PCR analysis of *OsLEA3* expression in 4‐week‐old genotypes after 7‐d deprivation of water. (C) Schematic diagrams showing effector and reporter constructs used in the transfection assays in D. (D) Relative activities of the *ProOsLEA3‐LUC* reporters co‐transformed with the different effector constructs in rice protoplasts. GFP and RFP effectors were used as negative controls. (E) Direct binding of OsDT2‐3FLAG to the *OsLEA3* promoter. The upper panel shows the schematic diagrams and amplified fragments in the *OsLEA3* promoter. Asterisks indicate the ACGT motifs in the promoter of *OsLEA3*. The lower panel shows the histogram revealing OsDT2 binding to the target promoter. Four‐week‐old seedlings were subjected to 7‐d dehydration and immediately collected for ChIP analyses. Chromatin was immunoprecipitated using IgG or anti‐FLAG antibody. The enrichment fold was calculated by normalizing the amount of the target DNA fragment against a genomic fragment of *Actin*, an internal control, and then shown as IP/Input. (F) ChIP analysis of OsDT2‐3FLAG association with *OsLEA3* chromatin in the absence or presence of OsbZIP66. (G) ChIP analysis of OsbZIP66‐9myc association with the chromatin of *OsLEA3* with or without OsDT2. (H) Overexpression of *OsbZIP66* increases OsDT2 binding capacity to the *OsLEA3* promoter. (I) Overexpression of *OsDT2* elevates OsbZIP66 association with the regulatory regions of *OsLEA3*. Different letters in A, B and D indicate significant differences (*p* < 0.05, one‐way ANOVA with Tukey's test). Asterisks in the bar graphs of E‐I indicate significant differences between the indicated groups (two‐tailed Student's *t*‐test, *, *p* < 0.05; **, *p* < 0.01; ***, *p* < 0.001). Error bars in this figure indicate means ± SD (*n* = 3). These experiments were repeated three times with similar results.

Among the 10 co‐regulated genes of OsDT2 and OsbZIP66, we focused on *OsLEA3*—a well‐characterized drought‐responsive gene previously reported as a direct target of OsbZIP66 [2, 4, 29, 31, 43]. To dissect how OsDT2 regulates *OsLEA3* expression under drought, we first examined its transcript levels in relevant genetic materials after 7 d of drought treatment. *OsLEA3* expression was consistently reduced in four independent *osdt2* mutant alleles but restored to wild‐type levels in two complementation lines (*osdt2‐1 gOsDT2‐3FLAG #3* and *#15*) (Figure 4A). Conversely, overexpression of *OsDT2* led to a continuous up‐regulation of *OsLEA3* (Figure 4B), indicating that OsDT2 positively regulates *OsLEA3* during drought response. These results are consistent with previous studies showing that *OsLEA3* enhances drought tolerance in rice [43]. Dual‐luciferase (LUC) reporter assays further showed that OsDT2 enhances the activity of the *OsLEA3* promoter (Figure 4C,D). To determine whether this regulation is direct, we performed chromatin immunoprecipitation (ChIP) using the *osdt2‐1 gOsDT2‐3FLAG* lines. The results revealed significant enrichment of OsDT2‐3FLAG at two specific genomic regions (fragments 5 and 6) of the *OsLEA3* locus (Figure 4E). Collectively, these data demonstrate that OsDT2 directly binds to the *OsLEA3* promoter and activates its transcription, establishing *OsLEA3* as a direct downstream target of OsDT2 in the drought response pathway.

Considering that OsDT2 interacts with OsbZIP66 and both possess DNA‐binding ability, we investigated how they co‐regulate *OsLEA3* expression. Dual‐LUC assays showed that co‐expression of OsDT2 and OsbZIP66 synergistically enhanced *ProOsLEA3* promoter activity, significantly beyond the effect of either protein alone (Figure 4D). Subsequent ChIP experiments revealed that loss of *OsbZIP66* function markedly reduced the binding of OsDT2 to the *OsLEA3* chromatin (Figure 4F). Similarly, disruption of *OsDT2* severely impaired OsbZIP66 binding to the *OsLEA3* promoter (Figure 4G). Furthermore, overexpression of *OsbZIP66* strengthened OsDT2 occupancy at the *OsLEA3* locus, and vice versa (Figure 4H,I). Together, these findings indicate that OsDT2 and OsbZIP66 mutually facilitate each other's binding to the *OsLEA3* promoter, thereby cooperatively enhancing its transcriptional activation under drought stress.

### 2.5 OsNF‐YC4 Interacts With OsDT2 and OsbZIP66 and Coordinates Their Binding to *OsLEA3*

**FIGURE 5:**
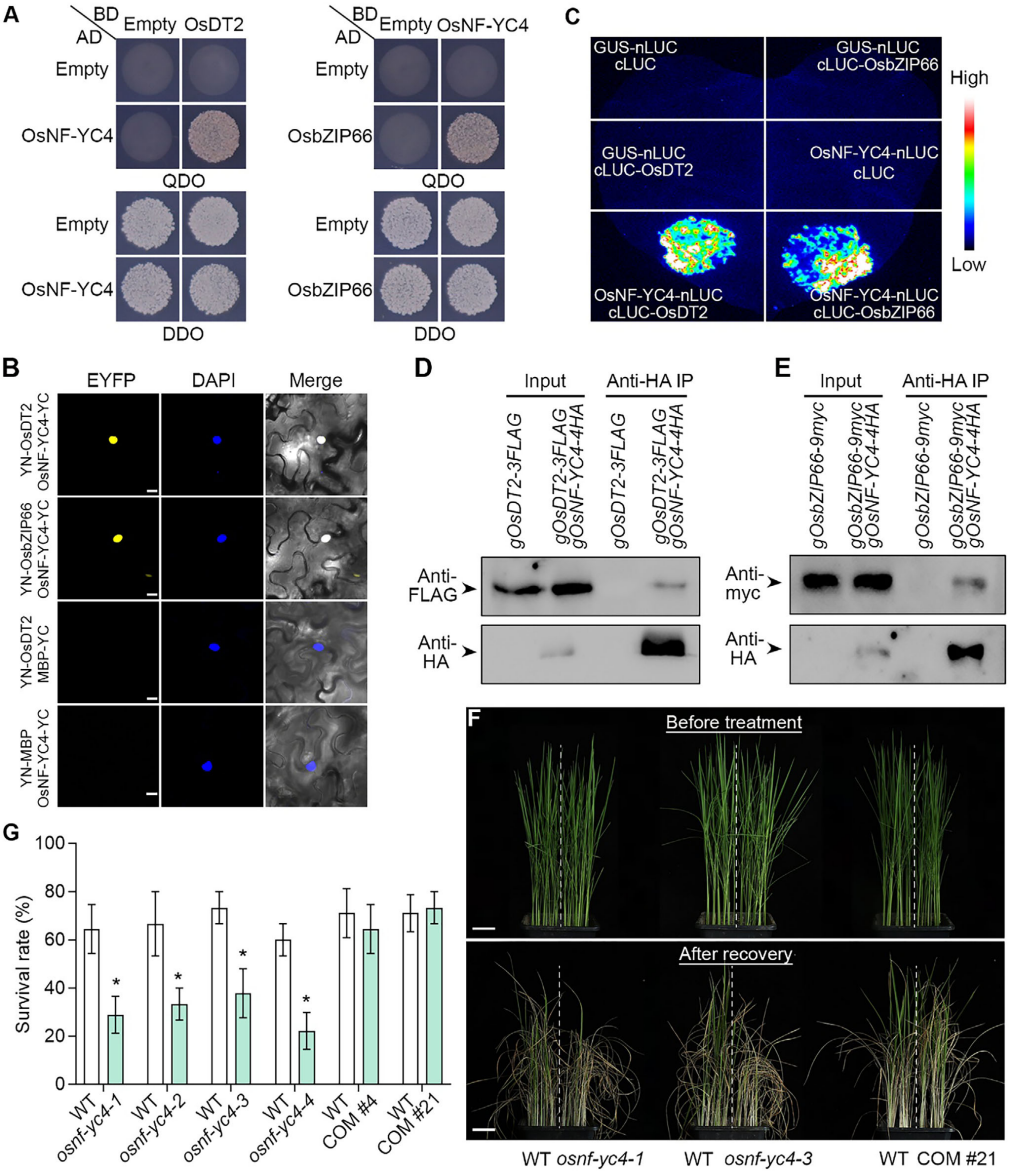
OsNF‐YC4 interacts with OsDT2/OsbZIP66 and positively regulates drought response in rice. (A) Y2H assay of interaction between OsNF‐YC4 and OsDT2 or OsbZIP66. (B) BiFC analysis showing the interactions among the indicated proteins. Scale bars, 10 µm. (C) LCI assay of interactions between OsNF‐YC4 and OsDT2 or OsbZIP66. (D and E) The in vivo interaction between OsNF‐YC4 and OsDT2 or OsbZIP66 is shown by CoIP assays. Total proteins were extracted from leaves of the indicated genotypes and incubated with anti‐HA magnetic beads. The input and coimmunoprecipitated protein were detected by anti‐FLAG, anti‐myc, or anti‐HA antibodies. (F and G) Drought resistance evaluation of wild type, *osnf‐yc4*, and COM (the complementation line, *osnf‐yc4‐1 gOsNF‐YC4‐4HA*). Asterisks denote significant differences between wild type and *osnf‐yc4* (two‐tailed Student's *t*‐test, *, *p* < 0.05). Error bars indicate means ± SD (*n* = 3 biological replicates). Scale bars, 5 cm.

To further elucidate how OsDT2 and OsbZIP66 regulate drought response in rice, we performed co‐immunoprecipitation coupled with liquid chromatography‐tandem mass spectrometry (LC‐MS/MS) using the *osdt2‐1 gOsDT2‐3FLAG* and *osbzip66‐1 gOsbZIP66‐9myc* tagged lines to identify their potential interacting proteins. Notably, peptides corresponding to OsNF‐YC4 were consistently detected in both pull‐downs (Figure S6A,B). Given the established role of NF‐YC proteins in plant development and stress responses [37, 39, 41, 44, 45], we selected OsNF‐YC4 for further validation. A series of protein‐interaction assays confirmed the physical association between OsNF‐YC4 and both OsDT2 and OsbZIP66. Yeast two‐hybrid analysis showed strong interactions in yeast cells (Figure 5A). BiFC in tobacco leaves revealed reconstituted YFP signals at the nuclear periphery when OsNF‐YC4‐cEYFP was co‐expressed with either nEYFP‐OsDT2 or nEYFP‐OsbZIP66, while controls showed no signal (Figure 5B). LCI experiments further supported these interactions in plant cells (Figure 5C). Finally, CoIP assays using transgenic rice protein extracts demonstrated that OsNF‐YC4 associates with OsDT2 and OsbZIP66 in vivo (Figure 5D,E). To directly test whether OsDT2, OsbZIP66, and OsNF‐YC4 can simultaneously associate, we performed yeast three‐hybrid (Y3H) and LCI assays. In the Y3H assay, yeast cells co‐expressing AD‐OsbZIP66 and BD‐OsDT2 exhibited markedly enhanced growth on selective media when OsNF‐YC4 was simultaneously expressed (Figure S6C). LCI experiments demonstrated that the interaction of OsDT2 and OsbZIP66 was remarkably enhanced with the introduction of OsNF‐YC4 (Figure S6D). Taken together, these results indicate that OsNF‐YC4 physically interacts with both OsDT2 and OsbZIP66 in vitro and in planta. To examine the role of OsNF‐YC4 in drought tolerance, we generated four independent *OsNF‐YC4* knockout mutants (*osnf‐yc4‐1* to *osnf‐yc4‐4*) using CRISPR‐Cas9 (Figure S6E). Under drought stress, these mutants displayed a more sensitive phenotype than the wild type, while complementation of *osnf‐yc4‐1* with *gOsNF‐YC4‐4HA* largely restored drought tolerance (Figure 5F,G), confirming that OsNF‐YC4 positively regulates drought resistance.

**FIGURE 6:**
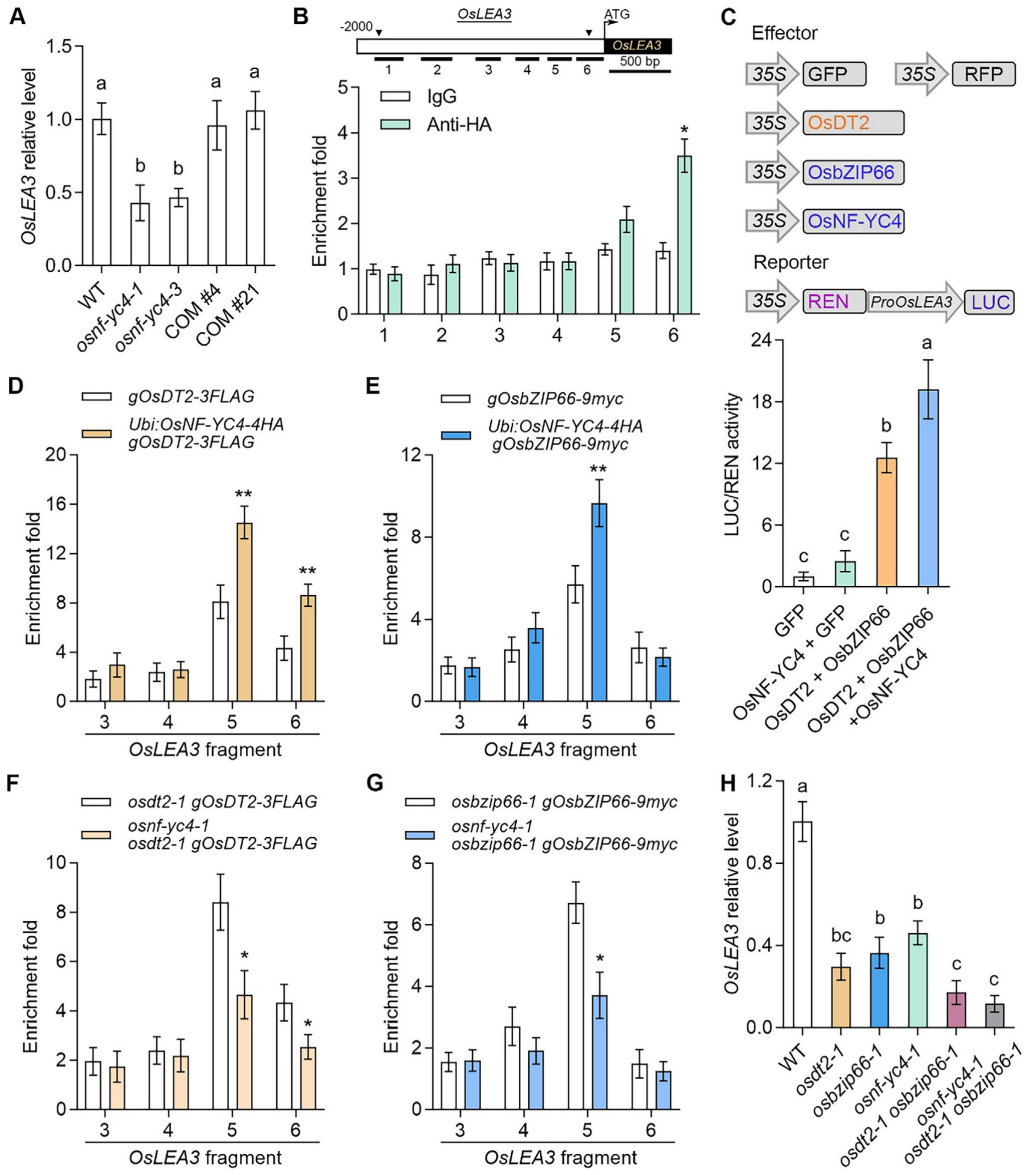
OsNF‐YC4 coordinates the binding of OsDT2 and OsbZIP66 to *OsLEA3* regulatory regions. (A) *OsLEA3* expression in genotypes after 7 d of water deprivation. (B) ChIP analysis of OsNF‐YC4 enrichment in *OsLEA3* promoter. Amplified fragments by qPCR are marked with 1‐6. Triangles indicate the CCAAT motifs in the promoter of *OsLEA3*. (C) The upper panel shows the constructs used in the transfection assay. The lower panels show the relative activities of *ProOsLEA3‐LUC* reporter in rice protoplasts co‐transformed with the indicated effector constructs. (D and E) Overexpression of *OsNF‐YC4* enhances OsDT2 or OsbZIP66 association with the regulatory regions of *OsLEA3*. (F and G) Disruption of *OsNF‐YC4* decreases OsDT2 or OsbZIP66 binding to *OsLEA3* chromatin. (H) Expression analysis of *OsLEA3* in 4‐week‐old genotypes after 7‐d drought treatment. Data are presented as means ± SD (*n* = 3 biological replicates). Asterisks indicate significant differences (two‐tailed Student's *t*‐test, *, *p* < 0.05; **, *p* < 0.01), and significant differences among genotypes indicated by different letters via one‐way ANOVA and Tukey's test (*p* < 0.05).

We next asked whether OsNF‐YC4 regulates the same set of downstream genes as OsDT2 and OsbZIP66. qRT‐PCR analysis showed that most previously identified co‐regulated genes, including the key marker *OsLEA3*, were down‐regulated in the *osnf‐yc4‐1* mutant (Figure S6F). Consistent with this, *OsLEA3* expression was reduced in all *osnf‐yc4* alleles but recovered in complementation lines (Figure 6A). Subsequent ChIP assays confirmed that OsNF‐YC4 directly binds to the *OsLEA3* promoter—particularly at fragment 6 (Figure 6B). The combinatorial effect of the three proteins was further examined. Notably, co‐expression of OsNF‐YC4 with both OsDT2 and OsbZIP66 resulted in substantially higher *OsLEA3* promoter activity than any individual protein or the OsDT2/OsbZIP66 pair alone (Figure 6C), indicating synergistic transactivation. This cooperativity was further supported by enhanced activation of *OsLEA3* by OsDT2 and OsbZIP66 upon addition of OsNF‐YC4 (Figure 6D,E). Conversely, loss of *OsNF‐YC4* markedly reduced the binding of both OsDT2 and OsbZIP66 to the *OsLEA3* chromatin (Figure 6F,G). These results demonstrate that OsNF‐YC4 facilitates the association of OsDT2 and OsbZIP66 with the *OsLEA3* promoter.

Consistently, *OsLEA3* expression under drought was significantly reduced in *osdt2‐1*, *osbzip66‐1*, or *osnf‐yc4‐1* single mutants and further suppressed in double and triple mutants (Figure 6H), confirming that all three proteins cooperate to regulate *OsLEA3*. Moreover, the ABA sensitivity was significantly reduced in single mutants of *OsDT2*, *OsbZIP66*, and *OsNF‐YC4* (Figure S7A,B), as reflected by their ABA‐insensitive shoot growth phenotypes. What's more, these mutants exhibited significantly higher germination rates than wild‐type seeds under ABA treatment, demonstrating their reduced sensitivity to ABA during seed germination (Figure S7C,D). These results further support the role of the OsDT2‐OsbZIP66‐OsNF‐YC4 module in ABA‐dependent drought signaling. At the physiological level, the mutants exhibited elevated accumulation of malondialdehyde (MDA) and H_2_O_2_ and decreased proline content under drought stress (Figure S8A–C), aligning with their drought‐sensitive phenotypes. Moreover, all three mutants exhibited higher water‐loss rates and more rapid declines in relative water content (RWC) compared to the wild type during dehydration (Figure S8D,E), indicating impaired water retention capacity. Taken together, these results demonstrate that OsDT2, OsbZIP66, and OsNF‐YC4 constitute a transcriptional module that co‐activates *OsLEA3* expression via enhanced promoter occupancy, thereby regulating ABA‐mediated drought tolerance and maintaining plant water status in rice.

### 2.6 DT2 is Functionally Conserved Across Plant Species

**FIGURE 7:**
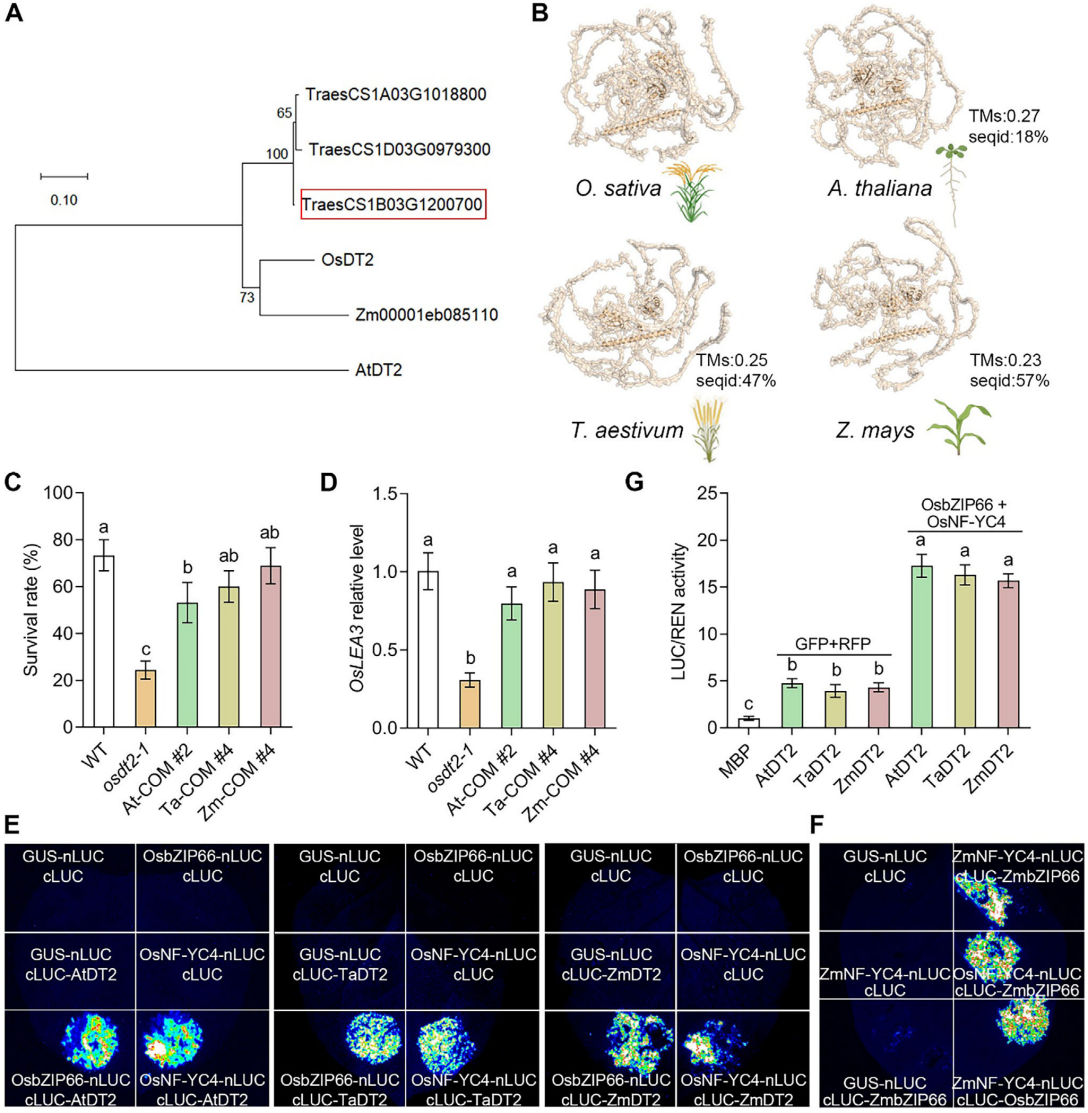
The DT2 function is conserved across several plant species. (A) Phylogenetic analysis of *OsDT2* and its orthologs. The evolutionary relationships among *OsDT2* and its orthologs from wheat, maize, and *Arabidopsis* were constructed using MEGA‐7 software. The gene highlighted in the red box was selected for further functional analysis in wheat. (B) The protein structures of OsDT2, AtDT2, TaDT2, and ZmDT2 modeled by the online AlphaFold prediction software. The corresponding template modeling scores (TMs) and sequence identity (SeqId) are shown below each predicted structure. (C) Survival rates of different genotypes after 14 d of water‐deficit and then 7 d of rewatering growth. (D) qRT‐PCR analysis of *OsLEA3* expression in 4‐week‐old wild type, *osdt2‐1*, and different COM lines after 7 d of water deprivation. (E) LCI assays demonstrating the interaction between AtDT2/TaDT2/ZmDT2 and OsbZIP66 or OsNF‐YC4. (F) LCI assays showing the interaction between ZmbZIP66 and ZmNF‐YC4, ZmbZIP66 and OsNF‐YC4 and OsbZIP66 and ZmNF‐YC4. GUS‐nLUC and cLUC were used as negative controls. (G) *ProOsLEA3*‐LUC activities induced by AtDT2/TaDT2/ZmDT2 in rice protoplasts co‐transformed with OsbZIP66 and OsNF‐YC4. MBP/GFP/RFP effector constructs served as the negative controls. Values in C, D, and G indicate means ± SD (*n* = 3 biological replicates). Different letters denote statistically significant differences (*p* < 0.05, one‐way ANOVA followed by Tukey's test).

Given the potential agricultural value of OsDT2, we investigated whether its function is conserved across other crops. Orthologs of OsDT2 were identified in *Arabidopsis* (*AtDT2*), wheat (*TaDT2*), and maize (*ZmDT2*). Phylogenetic and protein structure analyses revealed high similarity among these homologs (Figure 7A,B). Functional complementation assays showed that expression of *AtDT2*, *TaDT2*, or *ZmDT2* in the *osdt2‐1* mutant, driven by a 2.0‐kb 5’ upstream sequence of *OsDT2*, significantly restored drought tolerance (Figure 7C) and recovered *OsLEA3* transcript levels to near wild‐type values (Figure 7D), indicating conserved roles in drought response. Moreover, LCI assays confirmed that all three homologs physically interact with OsbZIP66 and OsNF‐YC4 (Figure 7E). Both intra‐ and interspecies interactions between bZIP66 and NF‐YC4 homologs from rice and maize were also detected (Figure 7F), suggesting that the interaction of this module is evolutionarily conserved across species. Dual‐LUC reporter assays further demonstrated that each homolog activated the *OsLEA3* promoter, and this activation was enhanced when OsbZIP66 and OsNF‐YC4 were co‐expressed (Figure 7G). Together, these results indicate that DT2 homologs from *Arabidopsis*, wheat, and maize share molecular and functional conservation with OsDT2, likely operating within a similar regulatory module involving bZIP66 and NF‐YC4 to enhance drought tolerance.

### 2.7 The DT2‐bZIP66‐NF‐YC4 Module is Conserved in Rice and *Arabidopsis* During Drought Response

**FIGURE 8:**
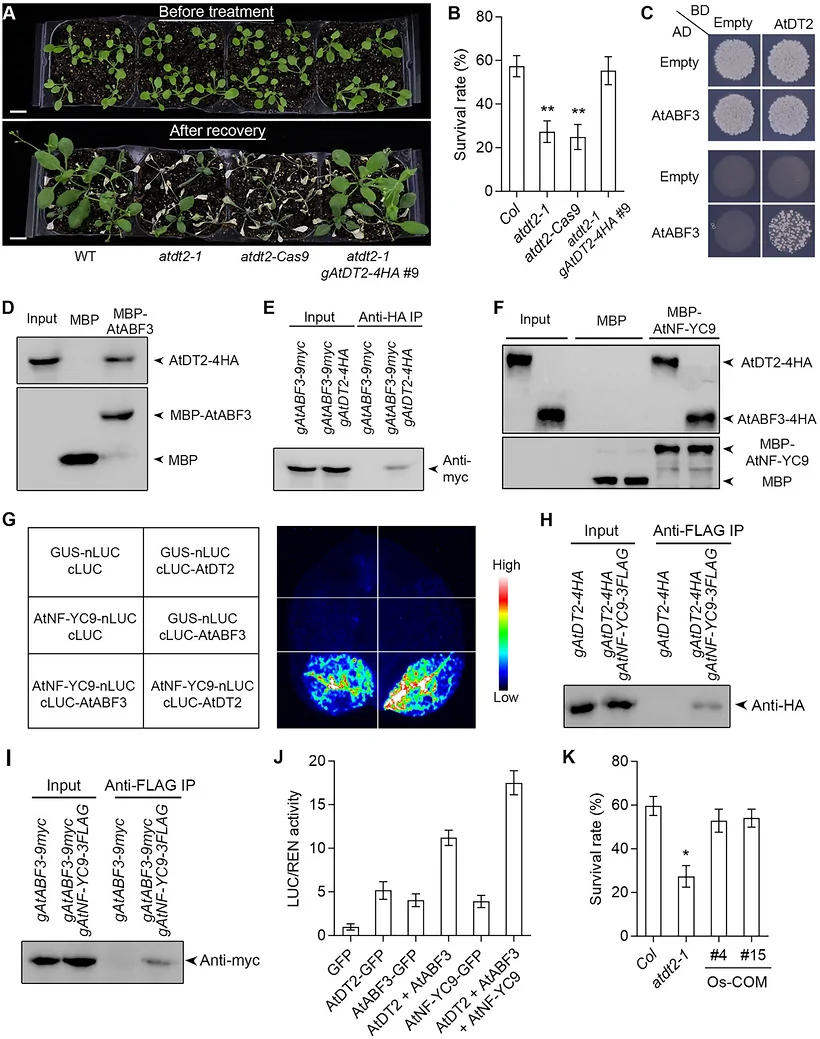
AtDT2‐AtABF3‐AtNF‐YC9 modulates drought tolerance in *Arabidopsis*. (A) Representative plants from the drought testing assay. Normally watered 2‐week‐old seedlings were subjected to dehydration treatment for approximately 10 d. The survival rates were examined after recovery for 7 d. Scale bars, 1 cm. (B) Survival rates of wild type, *atdt2* mutants, and *atdt2 gAtDT2‐4HA* after drought stress treatment. (C) Y2H assay of the interaction between AtDT2 and AtABF3. (D) In vitro MBP pull‐down assay of interaction between AtDT2 and AtABF3. HA‐tagged AtDT2 protein generated from *Arabidopsis* protoplasts was incubated with immobilized MBP or MBP‐AtABF3, respectively. Immunoblot analysis was performed using anti‐HA antibody. Input, 5% of the protein generated from *Arabidopsis* protoplasts. (E) CoIP experiment showing the in vivo interaction between AtDT2 and AtABF3 in *Arabidopsis*. The input and co‐immunoprecipitated proteins were checked using anti‐myc antibody. (F) In vitro pull‐down assay demonstrating AtNF‐YC9 interacts with AtDT2 and AtABF3. MBP‐AtNF‐YC9 was used as a bait, and pull‐down of AtDT2‐4HA or AtABF3‐4HA was detected by anti‐HA antibody. (G) LCI assays revealing the interaction between AtDT2 or AtABF3 and AtNF‐YC9. (H and I) The in vivo interaction between AtDT2 or AtNF‐YC9 and AtABF3 or AtNF‐YC9. (J) Relative activities of the *ProAtLEA3‐LUC* reporters co‐transformed with the different effector constructs. (K) Survival rates of wild‐type, *atdt2‐1*, and the complementation lines constructed by *gOsDT2*. Fourteen‐day‐old seedlings were subjected to drought stress by withholding water for 10‐12 d and recovered for 1 w. Values are means ± SD (*n* = 3 biological replicates). Significant differences were indicated by different letters (*p* < 0.05, one‐way ANOVA).

To assess the functional conservation of the DT2‐bZIP66‐NFYC4 module, we characterized the ortholog of *OsDT2* in *Arabidopsis thaliana*, *AtDT2* (AT3G27700). A T‐DNA insertion line (*atdt2‐1*) in the Col‐0 background was obtained, with the insertion located in the second exon (Figure S9A). In homozygous *atdt2‐1* plants, no full‐length *AtDT2* transcripts were detectable (Figure S9B). Under drought stress, *atdt2‐1* exhibited significantly reduced survival compared with the wild type (Figure 8A,B). This phenotype was further confirmed using an independent CRISPR‐Cas9 knockout line (*atdt2‐Cas9*), which also showed enhanced drought sensitivity (Figure 8A,B and Figure S9C). Genetic complementation with a genomic *AtDT2* fragment (*atdt2‐1 gAtDT2‐4HA*) fully restored drought tolerance (Figure 8A,B). Conversely, *AtDT2* overexpression lines displayed stronger drought resistance than the wild type (Figure S9D,E). Together, these results demonstrate that *AtDT2* acts as a positive regulator of drought response in *Arabidopsis*, supporting the evolutionary conservation of DT2 function in plants.

Since OsDT2 interacts with OsbZIP66 in rice, we next explored whether AtDT2 interacts with AtABF3, the ortholog of OsbZIP66 in *Arabidopsis* [27, 32, 46]. Y2H, pull‐down and CoIP assays showed that AtDT2 interacts with AtABF3 (Figure 8C–E). In addition, we identified *AT1G08970*, one of the most homologous genes to *OsNF‐YC4* in *Arabidopsis*, which has previously been named as *AtNF‐YC9* [47]. Pull‐down, LCI and CoIP experiments also confirmed the interaction between AtNF‐YC9 and AtDT2 or AtABF3 (Figure 8F–I). Collectively, these results substantiate that interactions among DT2, bZIP66, and NF‐YC4/9 are conserved in rice and *Arabidopsis*.

To genetically examine the interaction between AtDT2 and AtABF3, we generated the *atdt2‐1 atabf3‐1* double mutant. Under drought stress, the double mutant displayed a significantly stronger sensitive phenotype than either single mutant (Figure S9F,G). Consistent with this, the expression of *AtLEA3* was markedly reduced in all mutants, with the lowest level observed in the double mutant (Figure S9H). Subsequent ChIP assays confirmed that both AtDT2 and AtABF3 directly bind to the *AtLEA3* promoter. Specifically, AtDT2‐4HA was enriched at fragments 2 and 3, whereas AtABF3‐9myc bound selectively to fragment 2 (Figure S10A–C). To explore how they co‐regulate *AtLEA3*, we performed dual‐LUC assays. Co‐expression of AtDT2 and AtABF3 led to a significantly higher activation of the *AtLEA3* promoter than either protein alone (Figure 8J), indicating their cooperative transactivation. Furthermore, ChIP analysis revealed that the binding of AtABF3 to the *AtLEA3* promoter was notably reduced in the *atdt2‐1* background, and similarly, AtDT2 binding was diminished in *atabf3‐1* (Figure S10D,E), demonstrating that their association with chromatin is mutually reinforcing. Finally, to test functional conservation across species, we expressed the genomic *OsDT2* fragment in the *atdt2‐1* mutant. The transgenic plants showed drought tolerance comparable to the wild type, fully rescuing the mutant phenotype (Figure 8K). These results collectively indicate that DT2 orthologs function conservatively in drought response and cooperate with bZIP transcription factors to activate downstream targets.

**FIGURE 9:**
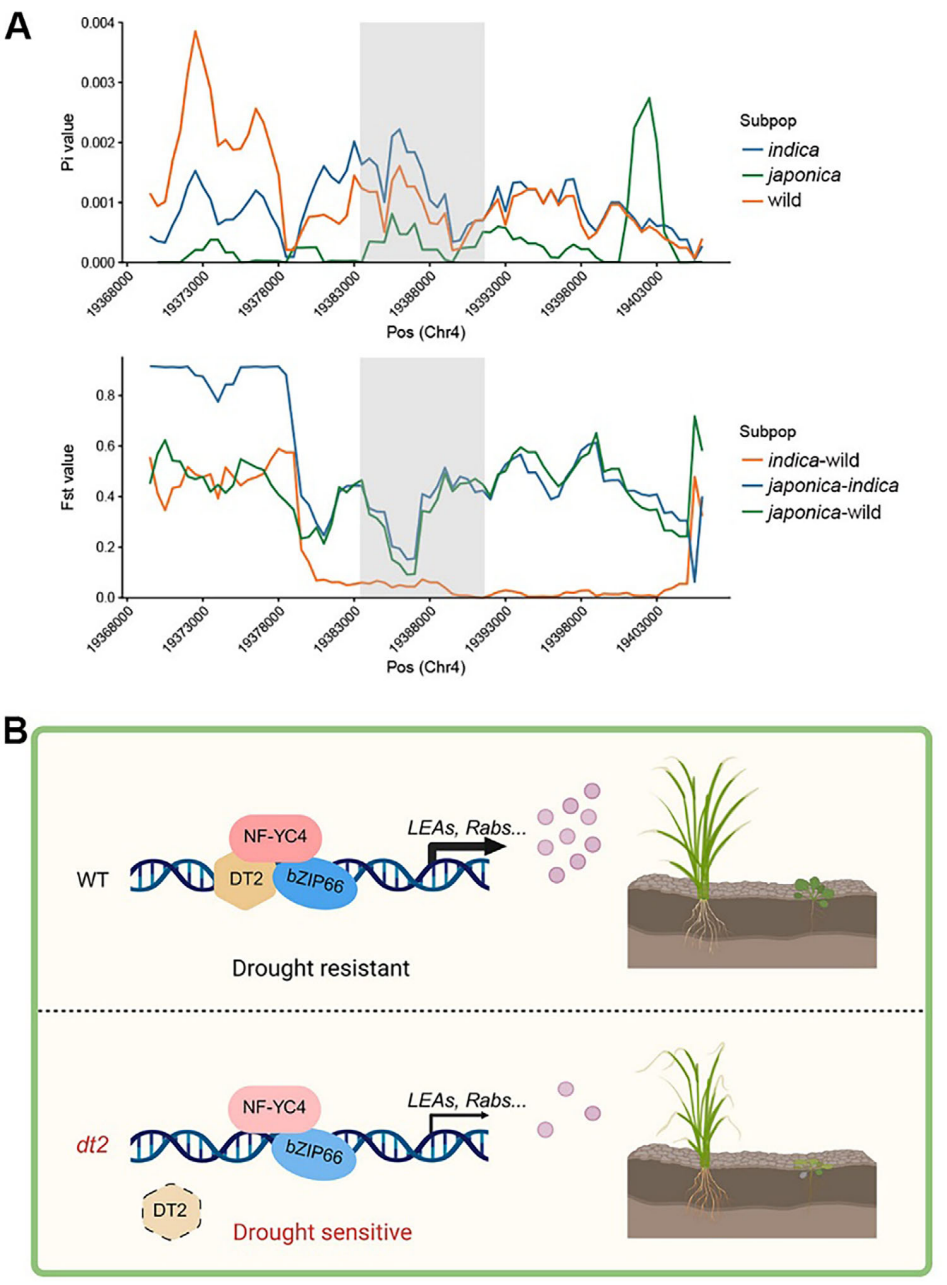
Natural variation around the *OsDT2* locus and a proposed working model for OsDT2‐mediated drought tolerance. (A) Nucleotide diversity (π) and pairwise population differentiation (Fst) across the genomic region surrounding *OsDT2* in *indica*, *japonica*, and wild rice groups. The shaded region indicates the *OsDT2* locus. (B) A proposed working model illustrating the role of the DT2‐bZIP66‐NF‐YC4 regulatory module in drought tolerance is as follows. Under drought stress in wild‐type plants, DT2 interacts with the transcription factors bZIP66 and NF‐YC4. These three proteins act cooperatively as a tripartite complex to activate the transcription of drought‐responsive genes, thereby enhancing water retention and drought tolerance. By contrast, in the *dt2* mutant, only a bZIP66‐NF‐YC4 dimer is formed. This dimer exhibits much lower transcriptional activity than the tripartite module, leading to reduced accumulation of osmoprotectants and consequently increased drought sensitivity in plants.

Given the positive role of OsDT2 in drought tolerance and its potential value for crop improvement, we further examined natural variation surrounding the *OsDT2* locus in different rice subpopulations. Analysis of nucleotide diversity (π) revealed distinct diversity patterns across this genomic region among *indica*, *japonica*, and wild rice groups. In particular, genetic diversity around *OsDT2* was highest in *indica*, intermediate in wild rice, and lowest in *japonica* (Figure 9A). Consistent with this pattern, pairwise Fst analysis showed clear population differentiation at this locus, with the strongest differentiation observed between *japonica* and *indica*, followed by that between *japonica* and wild rice, whereas differentiation between *indica* and wild rice was comparatively weak (Figure 9A). These results indicate that the genomic region surrounding *OsDT2* harbors substantial natural variation and exhibits marked subgroup differentiation, particularly between the two cultivated rice subspecies. Together, these findings suggest that *OsDT2* is a promising candidate for further allele mining and breeding applications in rice.

Based on the genetic, biochemical, and transcriptional evidence obtained in this study, we propose a working model for the role of the DT2‐bZIP66‐NF‐YC4 regulatory module in drought tolerance (Figure 9B). Under drought stress in wild‐type plants, DT2 interacts with bZIP66 and NF‐YC4, and these three proteins act cooperatively as a tripartite complex to activate the transcription of drought‐responsive genes such as *LEAs* and *Rabs*, thereby promoting osmoprotectant accumulation, enhancing water retention, and improving drought tolerance. By contrast, in the *dt2* mutant, the absence of *DT2* disrupts formation of the full tripartite module, leaving only the bZIP66‐NF‐YC4 dimer, which exhibits much lower transcriptional activity. As a result, downstream drought‐responsive gene expression is reduced, osmoprotectant accumulation is diminished, and plants become more sensitive to drought stress.

## 3 Discussion

In this study, we have identified OsDT2, encoding a CCCH‐type zinc finger protein, as a novel positive regulator of the drought stress response in rice. Our results further reveal that OsDT2, together with OsbZIP66 and OsNF‐YC4, form a high‐order complex to synergistically regulate drought‐related genes, such as *OsLEA3*, to respond to drought stress. This discovery provides a molecular explanation for the coordinated control of drought response in rice by a regulatory complex composed of transcription factors from diverse families and provides a target module for designing drought‐tolerant rice varieties.

Several lines of evidence support the essential role of OsDT2 in the drought response. First, loss‐of‐function OsDT2 mutants exhibit heightened sensitivity to drought, whereas overexpression of *OsDT2* enhances drought tolerance (Figure 1 and Figure S1), indicating that OsDT2 acts positively in drought signaling at the vegetative stage. More importantly, under drought stress throughout the entire growth cycle, *osdt2‐1* shows a significantly reduced seed‐setting rate and grain yield per plant, while *Ubi:OsDT2* plants display markedly increased values for both traits compared with the wild type (Figure 1E–G). These results demonstrate that upregulation of *OsDT2* improves reproductive performance under drought conditions, highlighting its potential for drought‐resistance breeding in rice. Notably, OsDT2 overexpression does not impose an obvious yield penalty under well‐watered conditions (Figure 1G), suggesting that its beneficial effects are primarily manifested under stress. This pattern suggests that OsDT2 functions as a context‐dependent regulator that enhances drought resilience without broadly promoting growth under all conditions. In addition, our population‐level analyses revealed clear natural variation and differentiation around the *OsDT2* locus among different rice subpopulations (Figure 9A). Furthermore, orthologs of OsDT2 from *Arabidopsis*, rice, wheat, and maize were able to substantially rescue the drought‐sensitive phenotype of *osdt2* mutants (Figure 7C,D). Phylogenetic and protein structure analyses revealed high similarity among these homologs (Figure 7A,B), indicating that OsDT2 is functionally conserved across monocots and dicots. This conservation provides compelling evidence for its fundamental role in drought adaptation.

Our data demonstrate that OsDT2 physically interacts with OsbZIP66, a bZIP transcription factor. This interaction appears to be functionally significant, as OsDT2 and OsbZIP66 not only share a common set of target genes but also act synergistically to promote the expression of key drought‐responsive genes (Figures 2 and 3). The regulatory network is further refined by the incorporation of OsNF‐YC4, a subunit of the conserved NF‐Y complex. Supported by Y3H and LCI assays, together with double and triple mutant analysis, we propose that OsDT2, OsbZIP66, and OsNF‐YC4 cooperatively assemble into an integrated transcriptional regulatory module controlling drought‐responsive gene expression (Figure 6I and Figure S6C,D). Consistent with this model, disruption of any of these components leads to increased water loss and reduced relative water content under drought conditions (Figure S8D,E), linking transcriptional regulation to physiological drought responses. A plausible mechanistic basis for this cooperativity is that these factors associate with spatially proximal cis‐elements within the *OsLEA3* promoter. OsbZIP66 likely recognizes ABRE/ABRE‐like elements, whereas OsNF‐YC4 is associated with nearby CCAAT‐containing regions [4, 37]. Together with our ChIP and interaction data, this supports a model in which OsDT2, OsbZIP66, and OsNF‐YC4 form a higher‐order complex on adjacent regulatory elements, thereby mutually stabilizing promoter occupancy and enabling coordinated activation of *OsLEA3* expression. Although a well‐defined consensus DNA‐binding motif for OsDT2 has not yet been established, our data demonstrate that OsDT2 is capable of associating with target promoters in vivo, suggesting that it directly contributes to DNA binding. The precise DNA‐binding characteristics of OsDT2 within this complex remain to be explored in future studies. Importantly, previous studies have demonstrated that *OsLEA3* overexpression improves drought resistance in rice under field conditions [43], which is consistent with our model that *OsLEA3* represents an important downstream effector of the OsDT2‐OsbZIP66‐OsNF‐YC4 module.

In canonical NF‐Y complexes, OsNF‐YC typically requires interaction with OsNF‐YB for nuclear translocation, where it associates with OsNF‐YA to form a heterotrimeric complex that recognizes the CCAAT *cis*‐element and regulates downstream gene expression [35, 48, 49, 50]. Here, we identified a non‐canonical module in which OsNF‐YC4 interacts with OsDT2 and OsbZIP66 to form a novel protein complex. This finding suggests an alternative mechanism for OsNF‐YC4 function, potentially independent of the classical NF‐YA/YB/YC trimer, or possibly acting in concert with it to fine‐tune transcriptional regulation under specific conditions, such as drought stress. However, the specific OsNF‐YA and OsNF‐YB subunits that may co‐participate with OsNF‐YC4 in this context remain unknown. Future studies employing yeast two‐hybrid screening or co‐immunoprecipitation coupled with mass spectrometry will be essential to identify the full complement of NF‐Y components associated with the OsDT2‐OsbZIP66‐OsNF‐YC4 module, thereby elucidating whether it represents a distinct complex or a variant of the canonical NF‐Y machinery.

Notably, we demonstrate that the OsDT2‐OsbZIP66‐OsNF‐YC4 module is evolutionarily conserved between monocots and dicots. The reciprocal functional complementation of drought‐sensitive phenotypes between *osdt2* and *atdt2* mutants by *AtDT2* and *OsDT2*, respectively (Figures 7C and 8K), provides strong genetic evidence for the conserved role of DT2 across species. Furthermore, the physical interaction among the *Arabidopsis* homologs AtDT2, AtABF3 (homolog of OsbZIP66), and AtNF‐YC9 (homolog of OsNF‐YC4) and synergistic effects on downstream target genes suggest that the core protein interaction network itself is preserved (Figures 4C–I, 6C–H, 7E–G, and 8C–J). This conservation underscores the fundamental importance of this regulatory module in plant drought adaptation responses.

In conclusion, our work unveils a conserved tripartite DT2‐bZIP66‐NF‐YC4 module that integrates signals from CCCH zinc finger proteins, bZIP transcription factors, and the NF‐Y complex to orchestrate the expression of drought‐protective genes. This discovery not only expands our understanding of the complex transcriptional networks underlying drought adaptation but also identifies promising synergistic genetic components for engineering crops with enhanced resilience to water scarcity. Future investigations focusing on the upstream signals that regulate this module and its downstream target repertoire will provide a more comprehensive view of its role in plant stress physiology.

## 4 Methods

### 4.1 Plant Materials and Growth Conditions

The rice cultivars used in this study were *Nipponbare* and ZH11, with the wild‐type implicitly referring to *Nipponbare*. For rice planting and seed propagation, all rice plants were grown in the experimental stations of the China National Rice Research Institute in Hangzhou, China. For drought treatment at the vegetative stage, rice was grown in a growth chamber (10 h light at 30°C/14 h dark at 25°C) at a relative humidity of around 70%. Light (500–700 nm, 100–200 µmol m^−2^ s^−1^) was provided by fluorescent white light‐tubes. For the *Arabidopsis thaliana* ecotype Col planting and seeds propagation, all *Arabidopsis* plants were grown on soil or Murashige and Skoog medium under LDs (16 h light/8 h dark) at 23°C ± 2°C.

### 4.2 Mutant Screening and Stress Treatment

The rice CRISPR/Cas9 RGKO‐ALL whole‐genome mutagenesis pool was obtained from the Biogle Genome Editing Center. Drought stress screening was conducted under natural field conditions at the Hainan experimental station. For each mutant line, approximately 100 seeds were directly sown in field plots (1 m length × 0.3 m width). Control lines were interspersed every 20 mutant lines for comparison, and all plants received uniform agronomic management. After 15 d of standard growth, irrigation was withheld to impose drought stress. After approximately 20 d of stress, leaf rolling rates were measured daily for one week.

For pot‐based drought treatment in rice, plants were arranged in a split‐plot design with 15 control and 15 transgenic/mutant plants grown together in black pots (18 × 18 × 12 cm^3^) filled with soil under normal growth conditions. Each experiment included six replicates per treatment. After four weeks of normal watering, irrigation was stopped for 12‐14 d. Survival rates were assessed following 7 d of re‐watering. For *Arabidopsis* drought treatment, plants were grown in black pots (54 × 28 × 53 mm^3^) containing vermiculite‐based soil under standard conditions, with six replicates per experiment. Two‐week‐old well‐watered seedlings were subjected to dehydration by withholding water for approximately 10 d. Drought tolerance was evaluated 7 d after re‐watering based on the resumption of growth, and survival rates were calculated accordingly. All drought tests were repeated at least four times.

The evaluation of key agronomic traits under drought stress, such as seed setting rate and yield per plant, was conducted over a minimum of three consecutive years in Hangzhou and Shanghai, China. Rice seeds were sown in batches under standard conditions in seedling nurseries. After 21 d, the seedlings were transplanted into rain‐sheltered fields and maintained under a water layer for 20 d. Thereafter, irrigation was withheld, and the plants were subjected to natural drying until seeds were harvested.

### 4.3 Plasmid Construction and Plant Transformation

To construct *pMKO‐Cas9‐osdt2/osnf‐yc4‐1/atdt2* vector, the *pMKO*‐Cas9 backbone was digested by *Bsa*I and ligated with the synthesized sgRNA oligos. A T‐DNA insertional mutant in the Col background was obtained from the *Arabidopsis* Biological Resource Center (http://www.arabidopsis.org). To create *Ubi:OsDT2‐3FLAG* construct, the coding sequence of *OsDT2* was amplified and inserted into Ubi‐pENTR‐3FLAG. To construct *gOsDT2‐3FLAG*, *gOsNF‐YC4‐4HA*, *gAtDT2‐4HA*, *gAtABF3‐9myc* and *gAtNF‐YC9‐3FLAG*, the gene bodies and upstream genomic sequences of *OsDT2*, *OsNF‐YC4*, *AtDT2*, *AtABF3* and *AtNF‐YC9* were amplified and integrated into pENTR‐3FLAG/4HA/9myc, respectively. To construct *ProOsDT2‐GUS*, the *OsDT2* promoter region used in *gOsDT2* was amplified and cloned into pENTR‐GUS. Transfer of the DNA fragment from the entry clone to pHGW by Gateway LR recombination reaction was performed according to the manufacturer's instructions (Invitrogen). Primers used for creating the above constructs are listed in Table S1. Transgenic rice plants were generated by *Agrobacterium*‐mediated transformation of rice calli as previously described [51]. All rice transgenic plants were selected by 50 µg/mL hygromycin. All Arabidopsis materials, including the mutants *atdt2‐1*, *atabf3‐1*, and the double mutant *atdt2‐1 atabf3‐1* are in the Col‐0 background. The transgenic plants were also generated in this background through *Agrobacterium tumefaciens*‐mediated transformation and selected on soil with Basta.

### 4.4 Physiological Measurements

Water loss rate (WLR) and relative water content (RWC) were measured using fully expanded leaves from 4‐week‐old, soil‐grown rice plants. For WLR measurement, leaves were excised and placed on paper under room conditions. Fresh weight (FW_t_) was recorded at the indicated time points (0‐4 h) after detachment, with the initial fresh weight defined as FW_0_. Water loss rate was calculated as the percentage of fresh weight loss relative to the initial weight using the following equation: WLR (%) = 100 × (𝐹𝑊_0_−𝐹𝑊_𝑡_)/𝐹𝑊_0_. For RWC measurement, leaves were collected from plants subjected to drought treatment at different time points (0, 3, 5, and 8 d). Fresh weights (FWs) were recorded immediately after sampling. The leaf samples were then immersed in distilled water with gentle shaking. After full rehydration, turgid weights (TWs) were measured following removal of surface moisture using tissue paper. The leaves were subsequently oven‐dried at 80°C for 24 h, and dry weights (DW) were recorded. Relative water content was calculated using the following equation: RWC (%) = 100 × (𝐹𝑊—𝐷𝑊)/(TW—DW).

### 4.5 Gene Expression Analysis

Total RNA was extracted using the Plant RNA Kit (Omega) and reverse‐transcribed to cDNA using Hifair III first Strand cDNA Synthesis SuperMix for qPCR (Yeasen). Quantitative real‐time PCR (qPCR) was performed on the Light Cycler 480 real‐time system (Roche) with the Hieff qPCR SYBR Green Master Mix (Yeasen). Each reaction was run in triplicate, and three biological replicates were included per experiment. *Actin* was used as the internal reference gene, and the relative expression levels were calculated as previously reported [4]. The primers used for gene expression analysis are listed in Table S1.

### 4.6 GUS Staining

For GUS staining, tissues from transgenic *ProOsDT2:GUS* plants were fixed in 90% acetone for 20 min at room temperature. After three washes with staining buffer [0.5 mM K_3_Fe(CN)_6_, 0.5 mM K_4_Fe(CN)_6_, 50 mM Na_2_HPO_4_, 50 mM NaH_2_PO_4_, pH 7.0], they were incubated in fresh staining buffer supplemented with 2 mM X‐Gluc (5‐bromo‐4‐chloro‐3‐indolyl‐β‐D‐glucuronide) and then subjected to vacuum infiltration for 15 min to ensure even substrate penetration. Subsequently, the samples were incubated at 37°C for 6 h to overnight. Finally, the stained tissues were decolorized in anhydrous ethanol to remove chlorophyll and clarify the GUS signal.

### 4.7 Yeast Two‐Hybrid Assay

The cDNA sequences encoding *OsDT2*, *OsNF‐YC4*, *AtDT2* and *AtABF3* were cloned into pGBKT7 or pGADT7 (Clontech). Yeast two‐hybrid assays were carried out using the Yeastmaker Yeast Transformation System 2 (Clontech). All yeast transformants were grown on SD/‐Trp/‐Leu or SD/‐Trp/‐Leu/‐His/‐Ade medium for the interaction test.

### 4.8 Pull‐Down Assay

To produce MBP‐tagged proteins, the coding sequences of *AtABF3* and *AtNF‐YC9* were individually cloned into the pMAL‐C_2_X vector (New England Biolabs). The expression of the empty pMAL‐C_2_X and recombinant proteins in *E. coli* Rosetta cells (DE3) (Novagen) was induced by IPTG at 37°C for 5 h. The soluble MBP fusion proteins were purified using amylose resin (New England Biolabs). For pull‐down assays, OsDT2‐3FLAG extracted from *Ubi:OsDT2‐3FLAG* and AtDT2‐4HA or AtABF3‐4HA generated from protoplasts were respectively incubated with the immobilized MBP and MBP‐fusion proteins at 4°C for 2 h. After three washes with binding buffer, proteins retained on the beads were resolved by SDS‐PAGE and detected by immunoblotting with anti‐FLAG antibody (Sigma), anti‐HA (Santa Cruz), or anti‐MBP (New England Biolabs) antibodies. The Super ECL Star Chemiluminescent Substrate (US Everbright) was used for signal detection.

### 4.9 BiFC Analysis

The full‐length coding regions of *OsDT2* and *OsNF‐YC4* were cloned into pGreen binary vectors harbouring C‐ and N‐terminal fusions of enhanced YFP to obtain *35S:nEYFP*‐*OsDT2*, *35S:OsDT2‐cEYFP* or *35S:OsNF‐YC4‐cEYFP* constructs. Equal amounts of plasmids were co‐transformed into *Agrobacterium* and then various combinations were co‐infiltrated into *N. benthamiana* leaves. Fluorescence signals were visualized using a confocal spectral microscope. *35S:nEYFP‐MBP* or *35S:MBP‐cEYFP* was used as a negative control.

### 4.10 LCI Assay

The coding regions of *OsDT2*, *OsNF‐YC4*, and *AtNF‐YC9* were fused with the N‐terminus of luciferase to generate *OsDT2‐nLUC*, *OsNF‐YC4‐nLUC*, and *AtNF‐YC9‐nLUC* constructs. Similarly, the cDNA sequences encoding *OsDT2*, *OsbZIP66*, *AtABF3* and *AtDT2* were fused with the C‐terminus of luciferase to obtain the *cLUC‐OsDT2*, *cLUC‐OsbZIP66*, *cLUC‐AtABF3*, and *cLUC‐AtDT2* constructs, respectively. All constructs were transformed into *Agrobacterium* and co‐infiltrated into 3‐week‐old *N. benthamiana* leaves. After 36–48 h of cultivation, the leaves were incubated with 0.5 mM D‐luciferin sodium salt substrate (Yeasen, 40901ES08) and kept in darkness for 5 min. Luciferase images were captured using a luminescence imaging workstation (Tanon 5200).

### 4.11 CoIP Experiment

Total proteins were extracted from crossed transgenic leaves with RIPA buffer (150 mM NaCl, 50 mM Tris‐HCl, pH 8.0, 1% Nonidet P‐40, 0.5% sodium deoxycholate, and 5 mM EDTA, pH 8.0) with freshly added 1 mM PMSF and 1 × protease inhibitor cocktail (Roche). The protein extracts were then incubated with anti‐FLAG beads (Sigma) or anti‐HA antibody (Sigma) at 4°C for 4 h. The total protein extracts as inputs and the immunoprecipitated proteins bound by the beads were resolved by SDS‐PAGE and detected with anti‐myc (Santa Cruz Biotechnology), anti‐FLAG (Sigma), or anti‐HA (Santa Cruz Biotechnology) antibodies. Immunoblots were performed using Super ECL Star Chemiluminescent Substrate (US Everbright).

### 4.12 Dual‐LUC Reporter Assay

The coding sequences of *OsDT2*, *OsNF‐YC4*, *AtDT2*, *AtABF3*, and *AtNF‐YC9* were cloned into pGreen‐35S or pGreen‐35S‐GFP vector to generate effector constructs. The 1‐kb promoter of *AtLEA3* was inserted into pGreen II 0800 LUC to create the reporter construct. Different combinations of effector and reporter plasmids (30 µg and 10 µg, respectively) were co‐ transfected into rice protoplasts. Firefly luciferase (LUC) and *Renilla* luciferase (REN) activities were examined using the dual‐luciferase reporter assay kit (Promega, E1960) according to the manufacturer's protocol. Relative luciferase activity was expressed as the LUC/REN ratio. 35S:GFP was included as a negative control. All experiments were performed with at least three biological replicates.

### 4.13 Seed Germination Assays

Freshly harvested seeds were first air‐dried under ventilated conditions for one week before germination assays. To detect the ABA sensitivity of seed germination, 100 seeds per genotype were soaked in 20 mL of distilled water or 2 µM ABA solution, which were then incubated in growth chambers at 28°C. The number of germinated seeds was recorded every 12 h. Germination was defined as the coleoptile reaching half the length of the seed. All phenotypes were consistently observed in at least three independent experiments, with 100 seeds per replicate. The germination rate was calculated as the number of total germinated seeds divided by the number of seeds spread.

### 4.14 RNA‐Seq Analysis

RNA‐seq analysis was conducted as previously described [4]. Briefly, total RNA samples were extracted from 4‐week‐old wild‐type and *osdt2‐1* subjected to a 7‐d drought treatment with three biological replicates each using TRIzol reagent (Invitrogen) according to the manufacturer's instructions. Sequencing libraries were prepared with the TruSeq PE Cluster Kit v3‐cBot‐HS (Illumina) and sequenced at Novogene Bioinformatics Technology (China). Raw sequencing reads were processed using Trimmomatic for quality filtering and then aligned to the rice reference genome. Gene expression levels were quantified with StringTie, and differential expression analysis was performed using DESeq2. Genes exhibiting a multiple‐test corrected *p*‐value of less than 0.05 were considered as differentially expressed. A heat map was generated via a cluster analysis tool to visualize gene expression patterns. GO enrichment analysis was performed using TBtools.

### 4.15 ChIP Assay

For the ChIP assay, samples of various genotypes deprived of water for 7 d were fixed in 1% formaldehyde under a vacuum, and the cross‐linking was quenched with 0.125 M glycine. Chromatin was then isolated and sonicated to generate DNA fragments with an average size of around 250 bp. The solubilized chromatins were immunoprecipitated using anti‐FLAG (Sigma), anti‐myc antibody (Sigma), or anti‐HA (Sigma) antibodies coupled to Protein A/G magnetic beads (Thermo Fisher). The immunoprecipitated DNA fragments were subsequently purified and analyzed by qPCR using the primers listed in Table S1.

### 4.16 Transient Expression of Proteins in Tobacco Cells and Rice Protoplasts

The coding region of *OsDT2* was cloned into pGreen‐GFP. *Agrobacterium*‐mediated transformation of *N. benthamiana* was carried out as previously described [4]. Fluorescent signals were observed under a confocal microscope 36‐48 h post‐infiltration. Rice protoplast isolation and PEG‐mediated transfection were performed following a previously established protocol [52]. After transformation, the protoplasts were incubated in darkness for 12‐16 h for further analysis.

### 4.17 Statistical Analysis

All data are presented as means ± SD, and the sample size (*n*) for each experiment is indicated in the corresponding figure legends. Bar charts and statistical analyses were generated using GraphPad Prism (version 9.5). In the qRT‐PCR analyses, results were first normalized against that of *Actin* and shown as the relative values to the corresponding control, which was set to 1 or 100%. In the ChIP assays, the enrichment fold was calculated by normalizing the amount of the target DNA fragment against a genomic fragment of *Actin*, an internal control, and then shown as IP/Input. Statistical significance between two sample groups was assessed using a two‐tailed Student's *t*‐test, with asterisks indicating levels of significance (*p < 0.05; **p < 0.01; ***p < 0.001). Comparisons among multiple sample groups were performed using one‐way ANOVA followed by Tukey's multiple comparisons test; different letters indicate statistically significant differences at *p* < 0.05.

## Author Contributions

J.S., Y.L., L.Z., X.M., and S.S. conceived the project and designed the experiments. J.S., Y.L., L.Z., X.M., C.P., C.Y., Y.W., K.Z., J.G., R.L., Z.Y., S.R., Y.H., X.L., and X.L. performed the experiments. J.S., Y.L., S.Z., M.L., P.Y., and S.S. conducted all statistical analyses. J.S., Y.L., L.Z., X.M., and S.S. analyzed the data. J.S., L.Z., and S.S. wrote the paper. All authors read and approved the manuscript.

## Funding

This work was supported by the National Natural Science Foundation of China (32441049 and 32322009), Natural Science Foundation of Zhejiang Province (LRG25C130001), Bio‐breeding Laboratory of Anhui Province (No. 2025SWYZ0100), and Zhejiang Key Laboratory of Crop Germplasm Innovation and Utilization Open Fund.

## Conflicts of Interest

The authors declare no conflicts of interest.

## Supporting information




**Supporting File**: advs76034‐sup‐0001‐SuppMat.docx.

## Data Availability

The data that support the findings of this study are available from the corresponding author upon reasonable request.
