# Supplementary material for: A Conserved DT2‐bZIP66‐NF‐YC4 Regulatory Module Confers Drought Tolerance in Rice and Arabidopsis
Source: Adv Sci (Weinh). 2026 Jun 9:e76034. Online ahead of print. doi: 10.1002/advs.76034 (PMC13337034; doi:10.1002/advs.76034)
Supplement: Supplementary file 1 — Supporting File: advs76034‐sup‐0001‐SuppMat.docx. [file ADVS-9999-e76034-s001.docx]

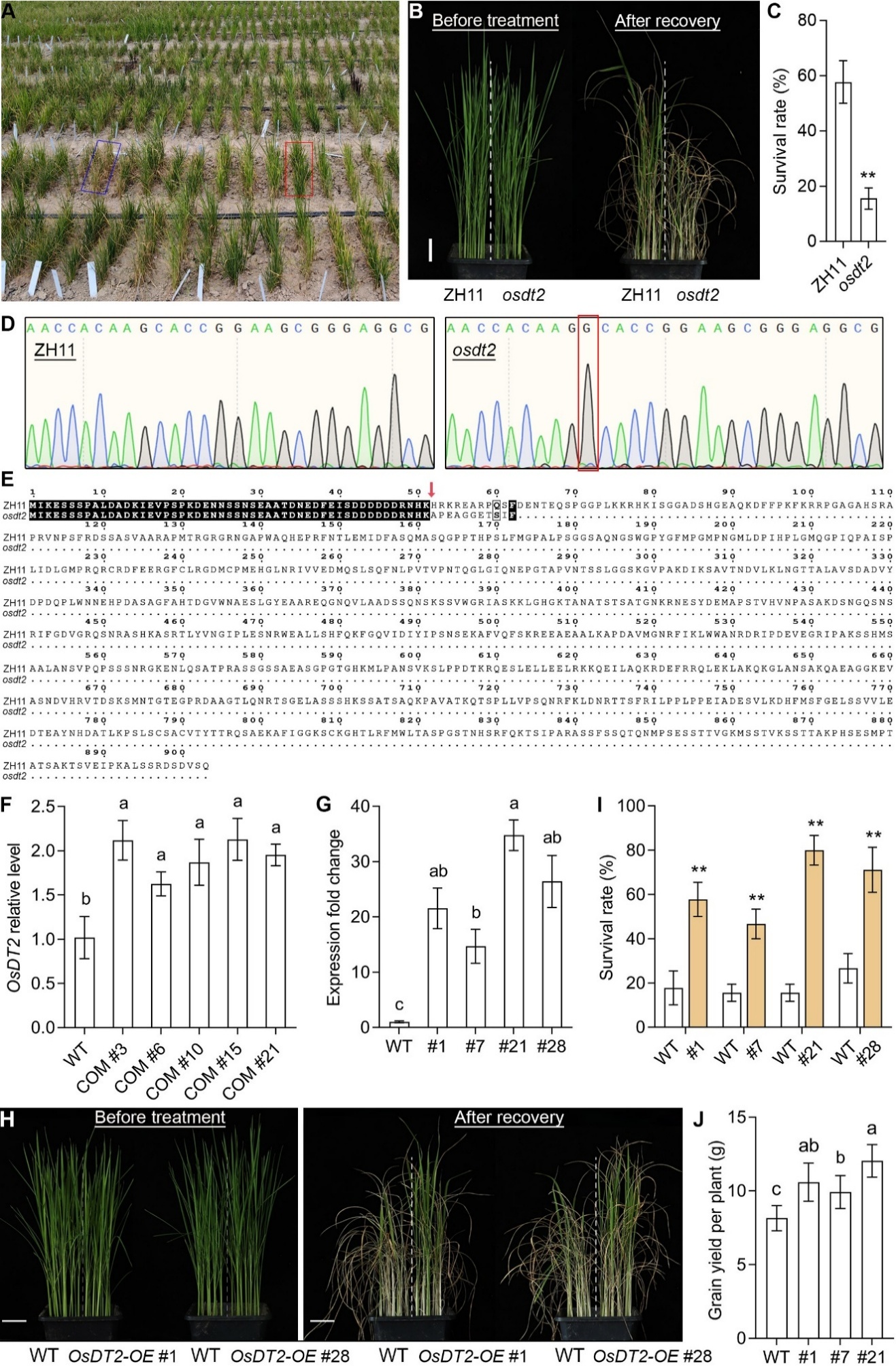


**Figure S1. OsDT2 positively regulates drought stress in rice.**

(A) Mutants obtained from the CRISPR/Cas9 mutant pool of rice were screened for drought sensitivity. The red box indicates wild type and the blue box indicates *osdt2* mutants.

(B and C) Phenotypes and survival rates of wild type and *osdt2* mutant planted in the soil before treatment or after recovery for 7 d from water deprivation. Scale bar, 5 cm.

(D) The detailed mutation position in the *OsDT2* genomic sequence in the *osdt2* mutant detected by sequencing.

(E) Sequence alignment of the DT2 protein between ZH11 and *osdt2*. The red arrow indicates the position of a G insertion, which alters the amino acid sequence and ultimately results in a premature stop codon.

(F) The expression levels of *OsDT2* determined by qRT-PCR in independent T2 *gOsDT2-3FLAG* lines. The levels of gene expression normalized to *Actin* expression are shown relative to the level in wild-type plants, which was set to 1.

(G) Expression fold change of *OsDT2* in independent *OsDT2-OE* (*Ubi:OsDT2-3FLAG*) lines. The expression level of *OsDT2* in the wild type was set to 1.

(H) Phenotype of wild type, *OsDT2-OE* #1 and #28 before drought treatment or after 1-w recovery from drought treatment. Scale bars, 5 cm.

(I) The survival rates of independent *OsDT2-OE* lines after recovery from dehydration treatment.

(J) Grain yield per plant of independent *OsDT2-OE* lines under DF. Error bars represent mean ± SD (*n* = 10 samples for each genotype).

Values in C, F, G and I are means ± SD (*n* = 3 biological replicates). Different letters in F, G and J denote significant differences (*P* < 0.05, one-way ANOVA followed by Tukey’s multiple comparisons test). Asterisks in C and I represent significant differences (two-tailed Student’s *t*-test, **, *P* < 0.01).

**
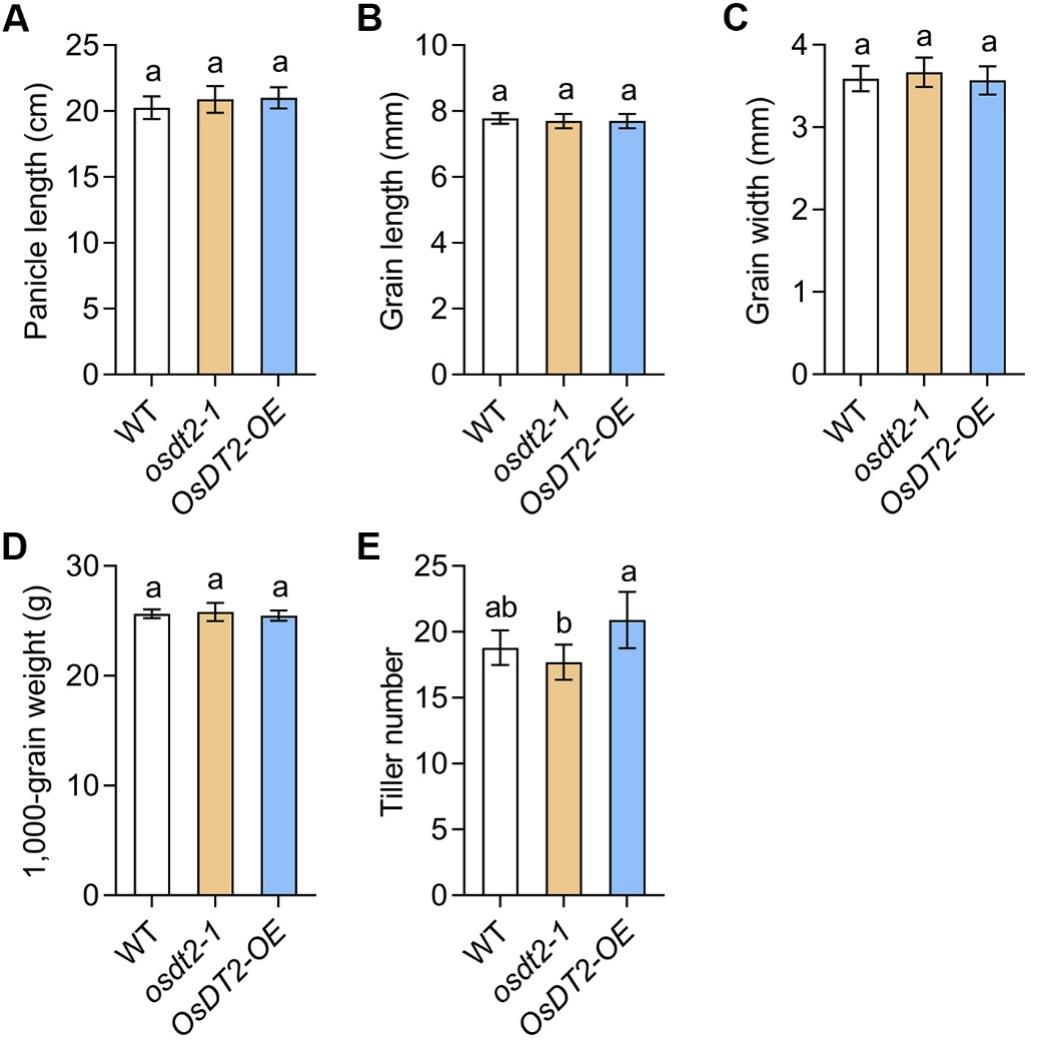
**

**Figure S2. Agronomic traits of wild type, *osdt2-1* and *OsDT2-OE* #28.**

(A to E) Analyses of *osdt2-1* and *OsDT2-OE* #28 of panicle length, grain length, grain width, 1,000-grain weight and tiller number. Data are the mean ± SD (*n* = 10 samples for each genotype). Statisticalsignificance determined by one-way ANOVA with Tukey’s test (*P* < 0.05).

**
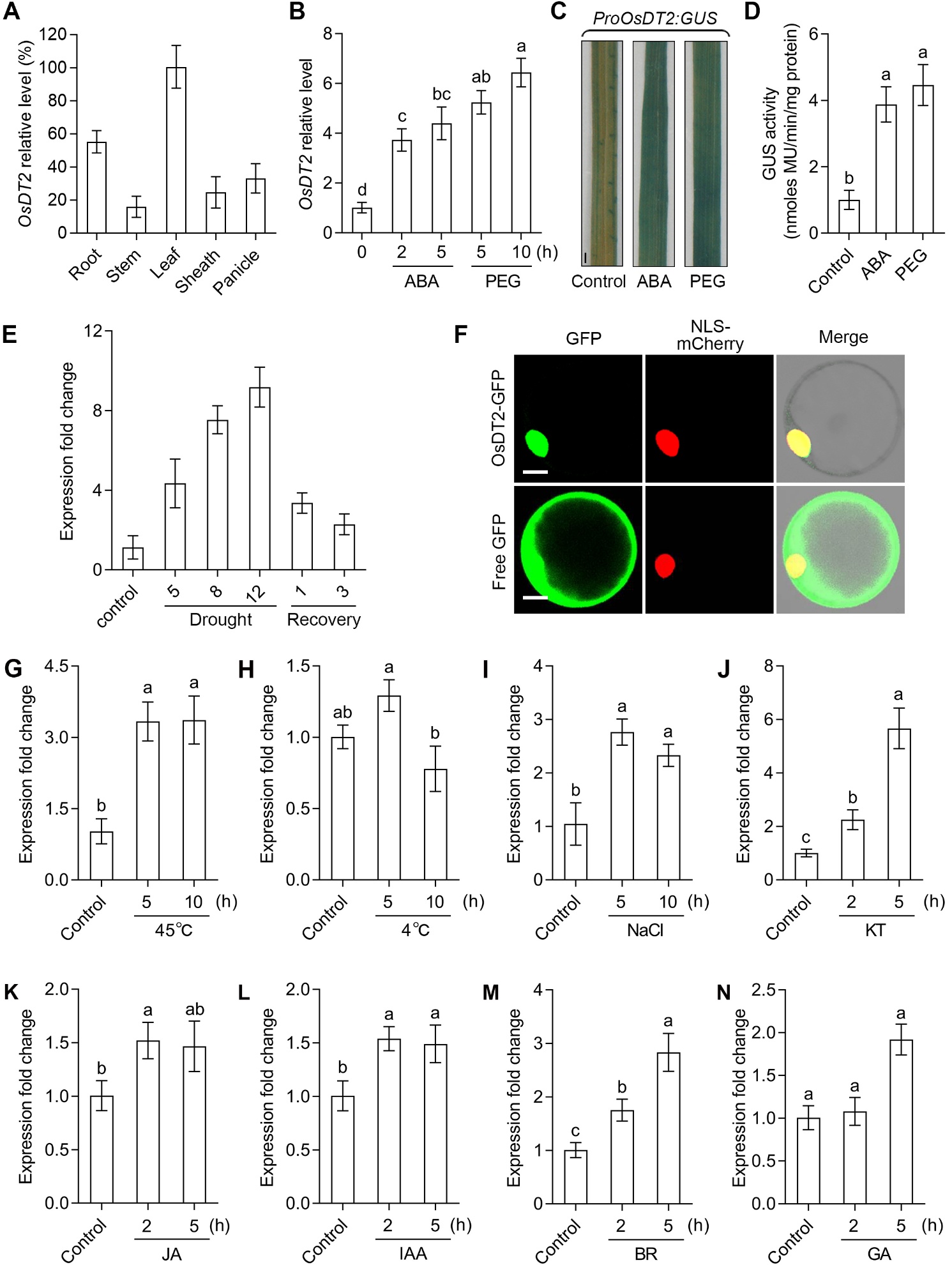
**

**Figure S3. Expression patterns of *OsDT2*.**

(A) qRT-PCR analysis of *OsDT2* expression in various wild-type tissues. Results were normalized against that of *Actin* and shown as the relative values to the highest expression set to 100%.

(B) Expression patterns of *OsDT2* under 0.1 mM ABA or 20% PEG6000 treatment. *OsDT2* expression was determined by qRT-PCR in wild-type plants collected at the indicated time points. The *OsDT2* level in seedlings without treatment was set to 1.0.

(C) GUS staining of *ProOsDT2:GUS* line in the leaves under ABA or PEG treatment. Scale bars, 1 mm.

(D) Quantitative comparison of GUS activities in control, ABA or PEG treatment.

(E) The *OsDT2* expression pattern under drought treatment. The levels of *OsDT2* expression was determined by qRT-PCR in wild-type plants collected at the indicated time points.

(F) The subcellular localization of OsDT2 in rice protoplasts. GFP, GFP fluorescence; NLS-mCherry, mCherry fluorescence of a nuclear marker; Merge, merge of GFP, mCherry and bright field. Scale bars, 10 μm.

(G to N) Expression patterns of *OsDT2* under high temperature, low temperature, salt or different hormone treatments. The relative gene expression levels were determined by qRT-PCR in wild-type plants collected at the indicated time points.

Values in A, B, D, E, and G-N are means ± SD (*n* = 3 biological replicates). Different letters in this figure indicate significant differences (*P* < 0.05, one-way ANOVA with Tukey’s test).


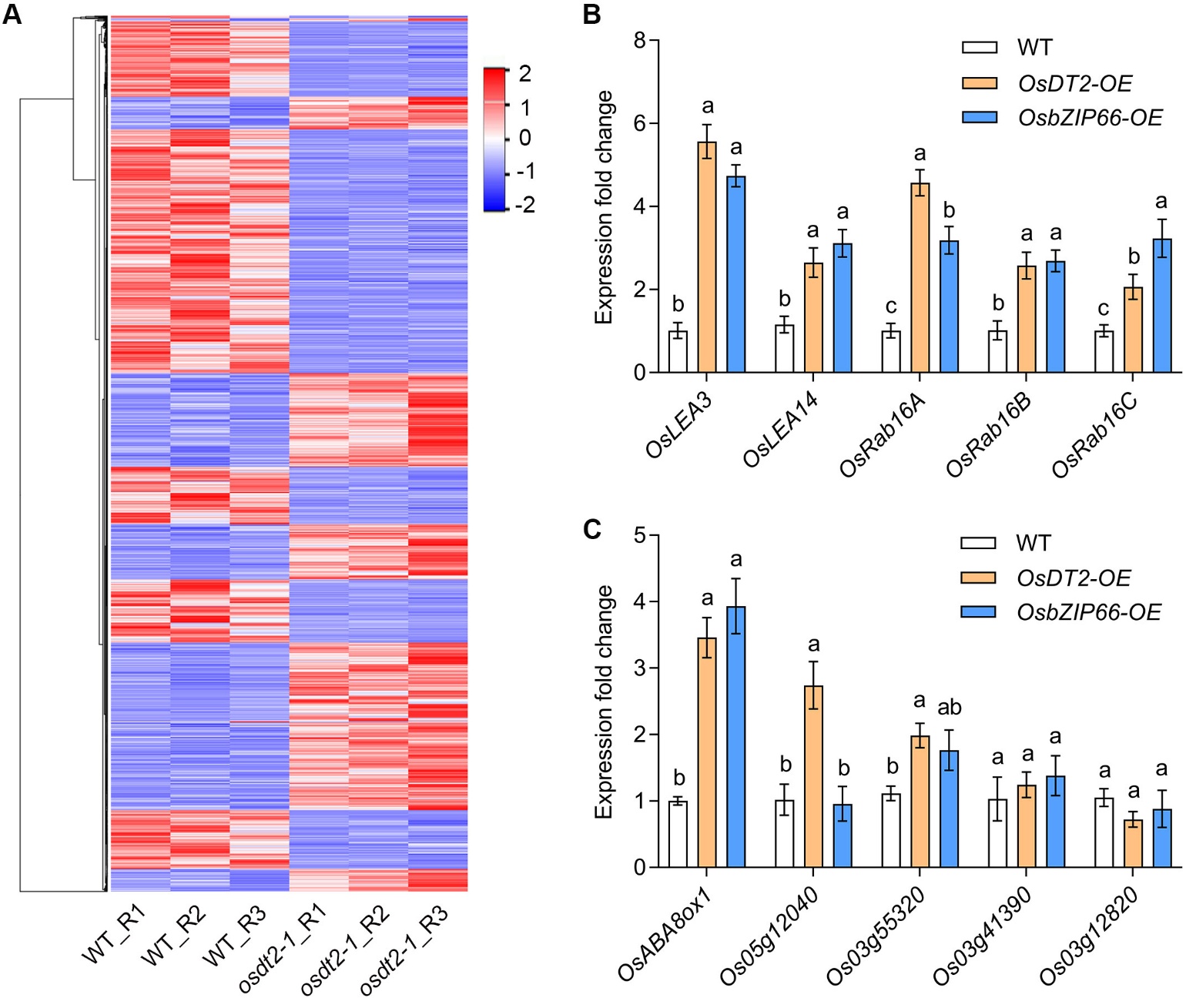


**Figure S4. OsDT2 and OsbZIP66 share a common set of downstream genes.**

(A) Heatmap of DEGs in *osdt2-1* compared with the wild type. Total RNA samples were extracted from 4-week-old seedlings treated under drought stress for 7 d with three biological replicates. The scale bar indicates fold changes (log_2_FC).

(B and C) qRT-PCR analysis of 10 stress-related genes in the *OsDT2-OE* #28 and *OsbZIP66-OE* #4 (*Ubi: OsbZIP66*) plants after 7 d of water deprivation. The experiments were repeated three times independently with similar results. Values are mean ± SD (*n* = 3 biological replicates). Significant differences indicated by different letters via one-way ANOVA and Tukey’s test (*P* < 0.05).


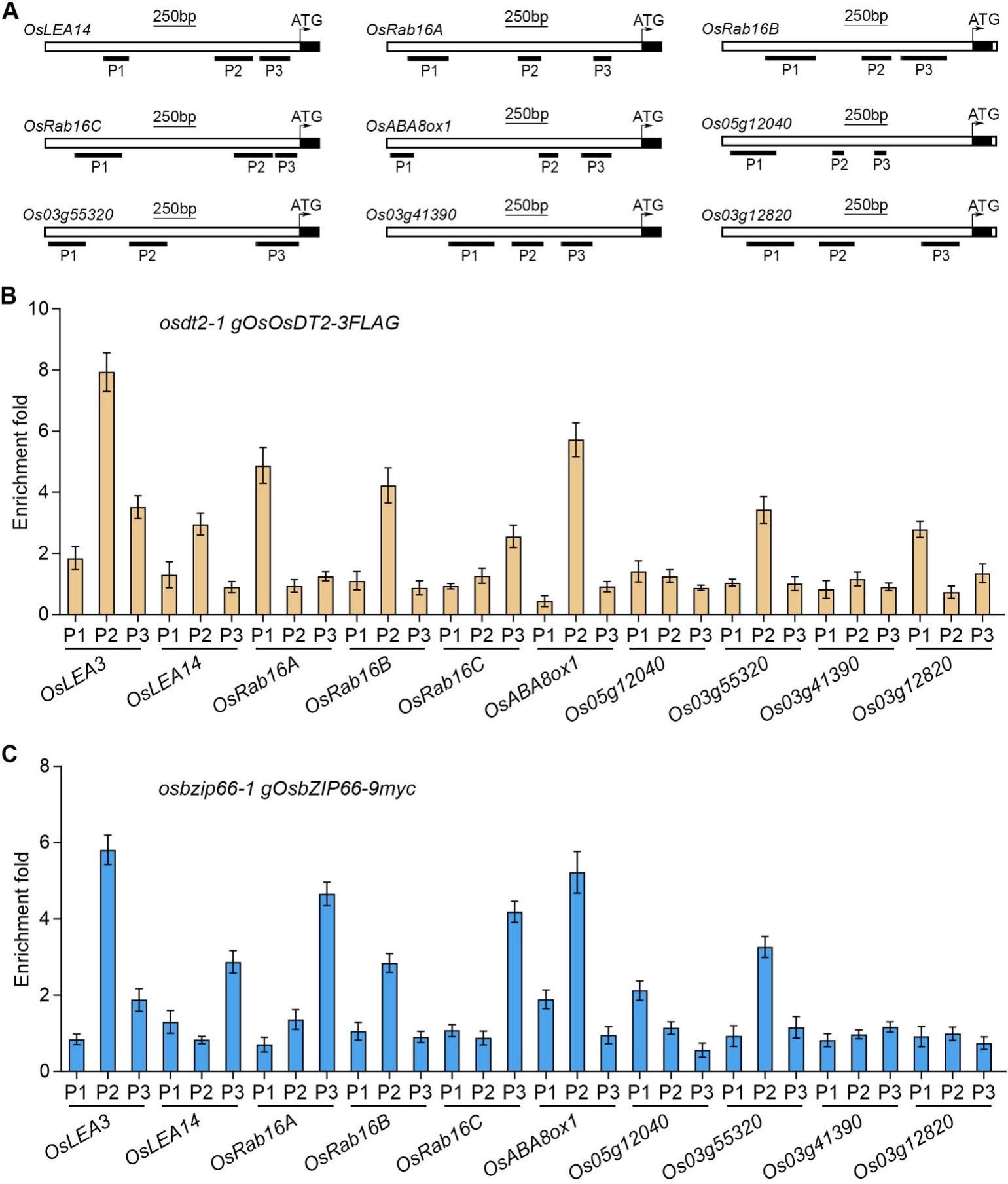


Figure S5. Direct binding of OsDT2 and OsbZIP66 to the promoters of 10 selected genes.

(A) Schemes show the promoter regions of the tested genes. The black lines indicate the amplified regions containing the consensus binding sequence of bZIP proteins.

(B and C) ChIP analysis of OsDT2 (B) or OsbZIP66 (C) binding to the promoters of tested genes. osdt2-1 gOsDT2-3FLAG, osbzip66-1 gOsbZIP66-9myc plants were treated for 7 d under drought stress and subsequently collected for ChIP analyses. Samples were precipitated by anti-FLAG, anit-IgG or anti-myc antibodies. The experiments were repeated three times independently with similar results. Data are mean ± SD (n = 3).

**
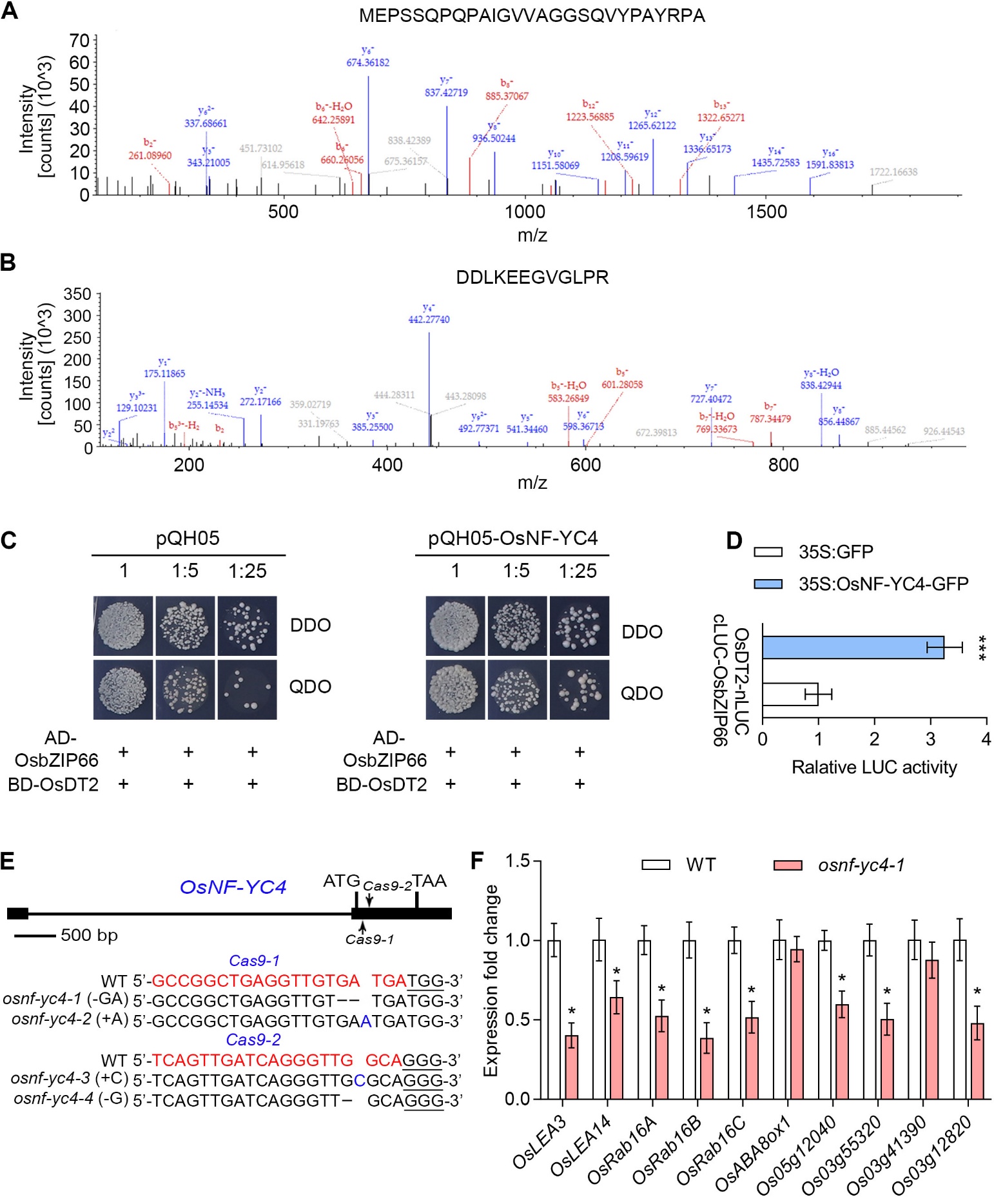
**

Figure S6. OsNF-YC4 was identified as a putative interacting partner of OsDT2 and OsbZIP66.

(A and B) The LC-MS/MS spectrum corresponds to an OsNF-YC4 peptide identified by CoIP using anti-FLAG magnetic beads or anti-myc antibody on total protein extracts from *osdt2-1 gOsDT2-3FLAG* or *osbzip66-1* *gOsbZIP66-9myc* plants. The identified OsNF-YC4 peptides are shown above the corresponding mass spectrum.

(C) Yeast three-hybrid assays show the interactions between OsDT2 and OsbZIP66 in the absence or presence of OsNF-YC4. The empty vector pQH05 was co-transformed with AD and BD plasmids to serve as a control in the left panel. Transformed yeast cells were grown on DDO and QDO medium.

(D) LCI assay of the effect of OsNF-YC4 on the interaction between OsDT2 and OsbZIP66. Values are means ± SD (*n* = 3 biological replicates). Asterisks indicate the significant difference (two-tailed Student’s *t*-test, ***, *P* < 0.001).

(E) CRISPR/Cas9-mediated target mutagenesis of *OsNF-YC4*. Exons and introns of *OsNF-YC4* are indicated by black boxes and lines, respectively. Alignments between wild-type and mutated sequences containing the target sites are shown below the schematic diagram. The target sequences adjacent to the underlined PAMs are indicated in red in wild-type sequences.

(F) Relative expression levels of 10 stress-related genes in *osnf-yc4-1* mutants detected by qRT-PCR. Values are means ± SD (*n* = 3 biological replicates). Asterisks indicate significant differences between wild type and *osnf-yc4-1* (two-tailed Student’s *t*-test, *, *P* <0.05).

**
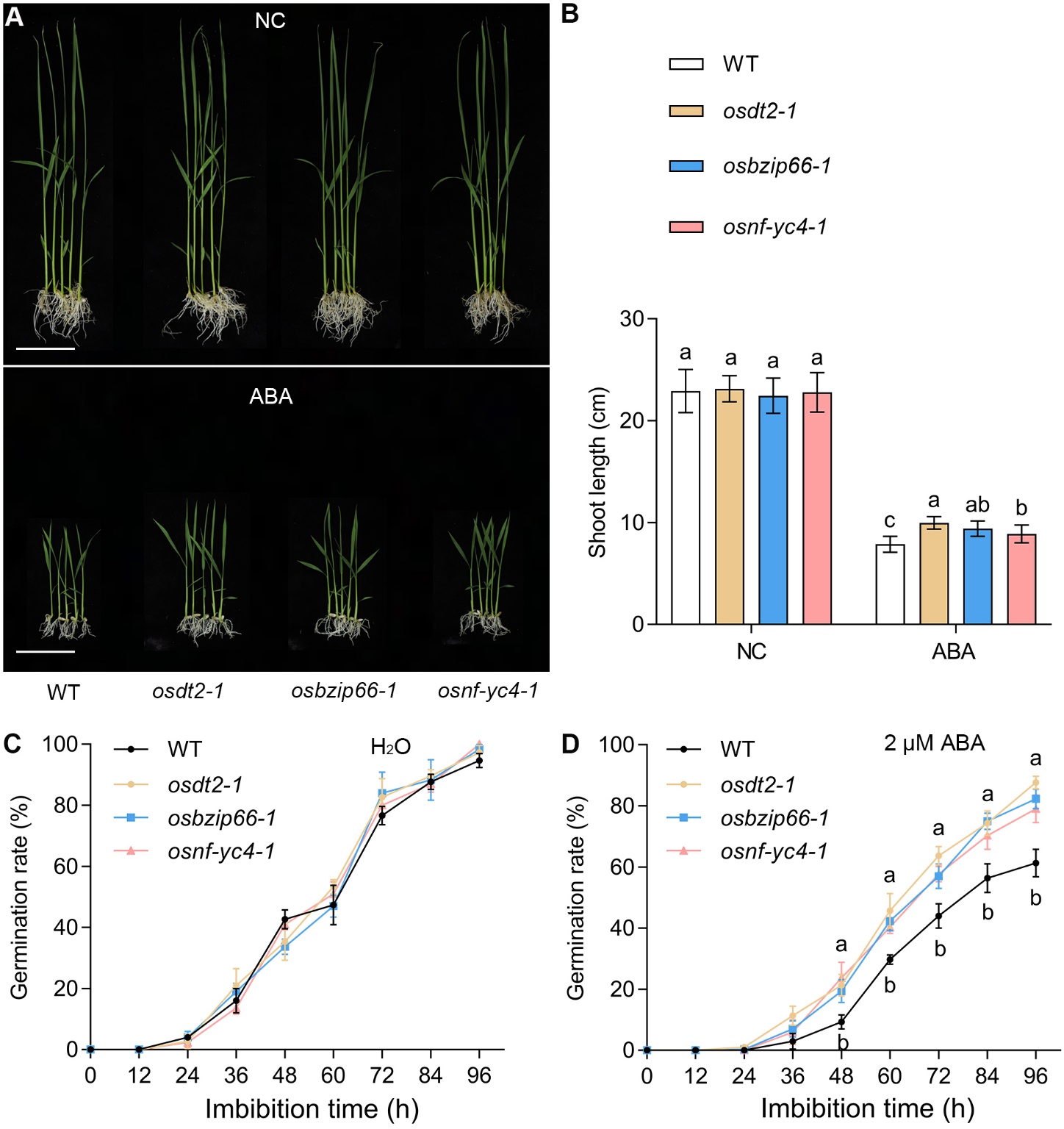
**

**Figure S7. Seed germination and seedling growth of OsDT2-OsbZIP66-OsNF-YC4 module in response to ABA.**

(A) Growth performance of wild type and various mutants on 1/2 MS medium containing 0 μM ABA (Normal conditions, NC) or 6 μM ABA (ABA treatment). Scale bars, 5 cm.

(B) The shoot length was measured at 10 d after germination shown in (A). Means ± SD of *n* = 15 samples for each genotype.

(C and D) Germination time courses of wild type and various mutants on 1/2 MS medium without ABA (C) or containing 2 μM ABA (D) for germination at 28 °C. Values are means ± SD (*n* = 3 biological replicates ).

Different letters represent significant differences determined by one-way ANOVA with Tukey’s test (*P* < 0.05).

**
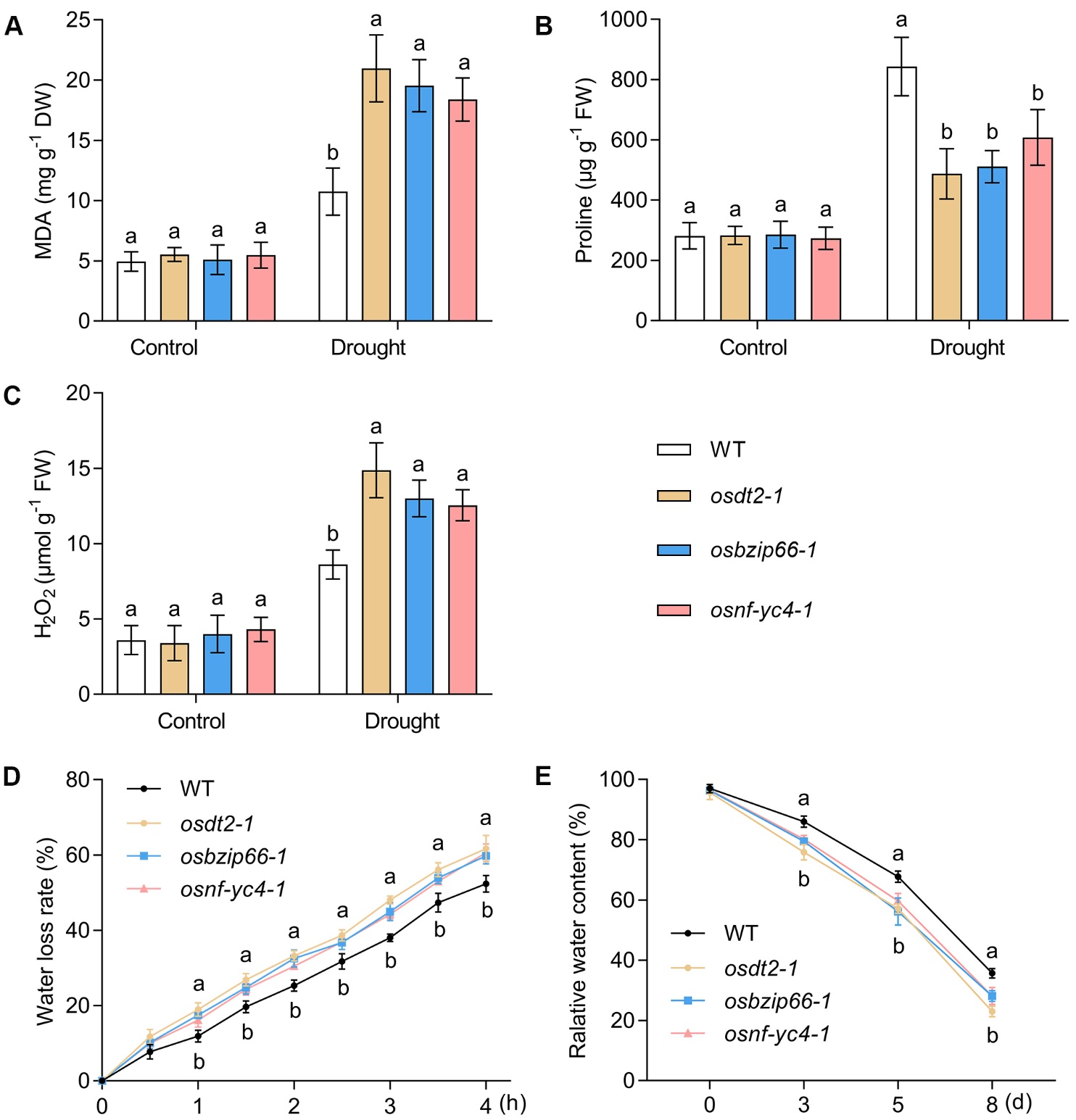
**

**Figure S8. The OsDT2-OsbZIP66-OsNF-YC4 module positively regulates drought signaling.**

(A to C) The MDA (A), Proline (B) or H_2_O_2_ (C) contents in various genotypes after 10-d drought treatment.

(D) Water loss in leaves of wild-type, *osdt2-1*, *osbzip66-1* and *osnf-yc4-1* mutants were performed within 4 h.

(E) Relative water content (RWC) in leaves of various genotypes under drought stress.

Data in A-E are presented as means ± SD (*n* = 3 biological replicates). Different letters indicate significant differences determined by one-way ANOVA followed by Tukey’s test (*P* < 0.05).

**
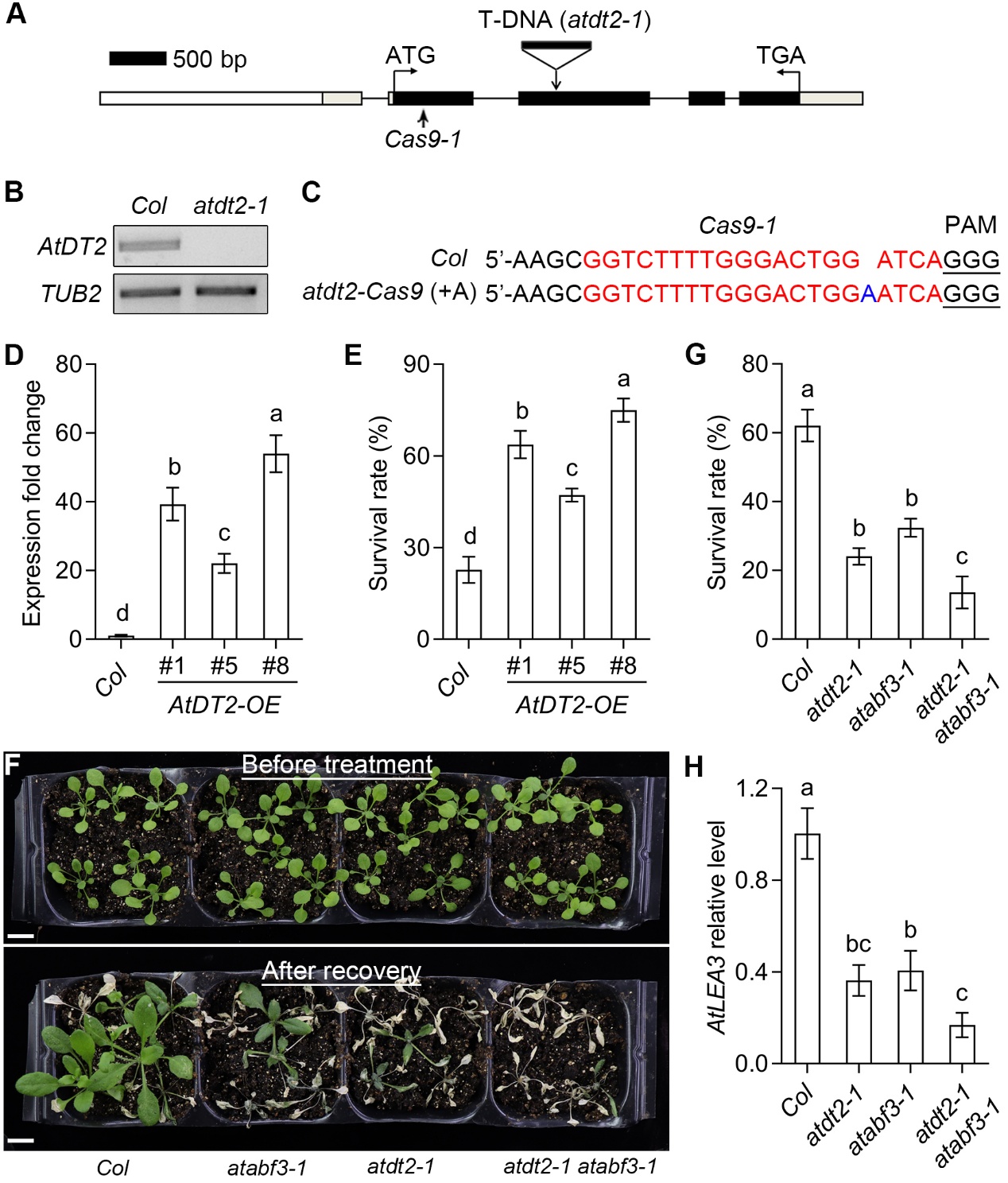
**

**Figure S9. *AtDT2* positively regulates drought response in *Arabidopsis*.**

(A) Schematic diagram shows the transfer DNA (T-DNA) insertion site in *AtDT2* and the target site of the Cas9 in *AtDT2-Cas9*.

(B) Full-length *AtDT2* transcript is undetectable in *AtDT2*. *TUB2* serves as the internal control.

(C) Alignments between wild-type and mutated sequences of *atdt2-Cas9* containing the target sites.

(D) Expression fold change of *AtDT2* in independent *AtDT2-OE* (*Ubi:AtDT2-4HA*) lines.

(E) The survival rates of independent *AtDT2-OE* lines after recovery from dehydration treatment.

(F) Drought phenotype of wild type, *atdt2-1*, *atabf3-1* and *atdt2-1 atabf3-1*. 14-d seedlings were subjected to 10-12 d of drought stress and recovered for 1 w. Scale bar, 1 cm.

(G) Survival rates of wild type, *atdt2-1*, *atabf3-1* and *atdt2-1 atabf3-1* mutants after recovery from water deprivation.

(H) qRT-PCR analysis of *AtLEA3* expression in 2-week-old wild type, *atdt2-1*, *atabf3-1* and *atdt2-1 atabf3-1* after water deprivation.

Error bars in D, E, G, and H indicate means ± SD (*n* = 3). Different letters denote statistically significant differences determined by one-way ANOVA with Tukey’s test (*P* < 0.05).

**
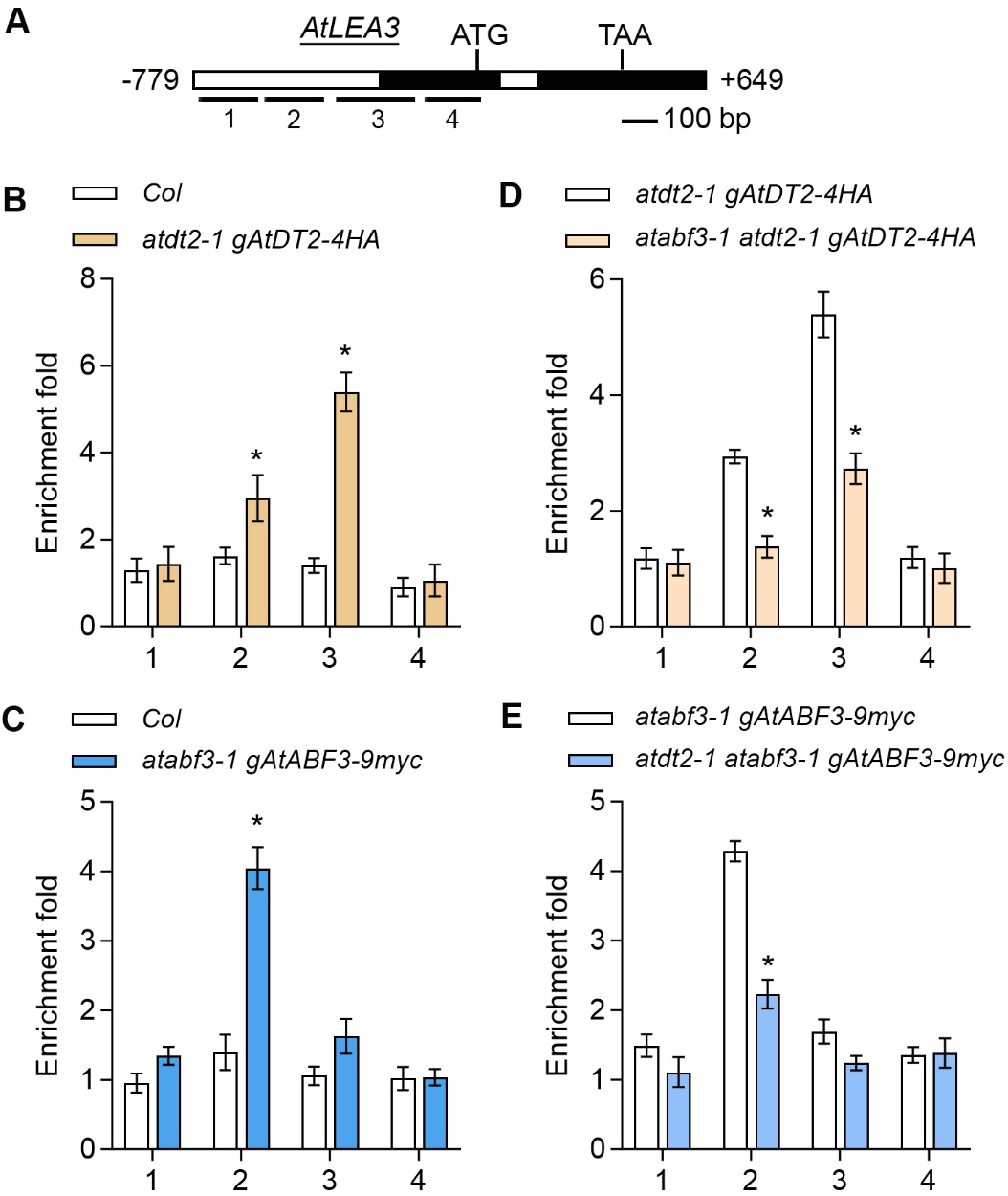
**

**Figure S10. ChIP analyses of AtDT2 and AtABF3 binding to the regulatory regions of *AtLEA3*.**

(A to C) ChIP analysis of AtDT2 or AtABF3 binding to the genomic regions of *AtLEA3*. The genomic structure of *AtLEA3* was indicated by black and white boxes.

(D) ChIP analysis of AtDT2-4HA binding to *AtLEA3* promoter in the presence or absence of AtABF3.

(E) ChIP analysis of AtABF3-9myc binding to the *AtLEA3* promoter with or without AtDT2.

In B-D, asterisks denote significant differences between the indicated pairs (two-tailed Student’s *t*-test, *, *P* < 0.05). Error bars represent means ± SD (*n* = 3). These experiments were repeated three times independently with similar results.

**Table S1. List of primers used in this study.**

| Gene Primers | |
| --- | --- |
| Primers for qRT-PCR | |
| *OsDT2-F* | 5'-GGTTAACAGCCTCTCCTGGT-3' |
| *OsDT2-R* | 5'-CTGTGAGGCTTTGCAGTTGT-3' |
| *OsNF-YC4-F* | 5'-CATCACCAGGACGGATAT-3' |
| *OsNF-YC4-R* | 5'-TGACCACCATACGCTATT-3' |
| *OsLEA3-F* | 5'-TTTCTGACGGGTGTGGGTGATG-3' |
| *OsLEA3-R* | 5'-AACACAGACGAGAAACTCTGACG-3' |
| *OsRab16A-F* | 5'-AGCTCAAGCTCGTCTGAGGATG-3' |
| *OsRab16A-R* | 5'-GGGAGCTTCTCCTTGATCTTCTCC-3' |
| *OsLEA14-F* | 5'-AGCGACGACGACAGATTAACCG-3' |
| *OsLEA14-R* | 5'-CCAGCTGCGCCATTGTTTCTTG-3' |
| *OsABA8ox1-F* | 5'-ACGTGGAATACCAAGGGTACCTG-3' |
| *OsABA8ox1-R* | 5'-TGTTGCGGAACAGAGGTAGCAC-3' |
| *OsRab16C-F* | 5'-AGCGTGAAATGCGAGTGATGGTG-3' |
| *OsRab16C-R* | 5'-ACGCGAGAACACAACAGACAATG-3' |
| *OsRab16B-F* | 5'-TTCGCACGTGTTGGGTTGTACC-3' |
| *OsRab16B-R* | 5'-ACACACACACCATACAAAGCTTGC-3' |
| *Os05g12040-F* | 5'-TATCATGAAGCCGGCGAAGCTG-3' |
| *Os05g12040-R* | 5'-CCATCTTGCAAAGTAACCCTCCAC-3' |
| *Os03g55320-F* | 5'-ACGGGTATAGCAAACAGATTGGTG-3' |
| *Os03g55320-R* | 5'-TCAGATCACGGAAGCTGACCTC-3' |
| *Os03g41390-F* | 5'-GTCTACTCATCCTTGCTGCAACTG-3' |
| *Os03g41390-R* | 5'-AGACCAGCTCGCCTGAATCTAC-3' |
| *Os03g12820-F* | 5'-GGACGTACAACTGCTTCAAGAGAG-3' |
| *Os03g12820-R* | 5'-GCAAGAACCGGATGAACTGGTC-3' |
| *OsActin-F* | 5'-AAGACTGTAATACCTATTG-3' |
| *OsActin-R* | 5'-ATAGAATAATCGCAACTC-3' |
| *OsUbq-F* | 5'-CTTTCAGCTGAGGCCCAAGA-3' |
| *OsUbq-R*  *AtLEA3-F*  *AtLEA3-R*  *AtTUB2-F*  *AtTUB2-R* | 5'-ACGATTGATTTAACCAGTCC-3'  5'-AAACCACAATGGCTCGTTCTCTCG-3'  5'-TTTCGGAGCCGAAGACGGATTG-3'  5'-ATCCGTGAAGAGTACCCAGAT-3'  5'-TCACCTTCTTCATCCGCAGTT-3' |
| Primers for constructs used for plant transformation | |
| *OsDT2-Cas9-1-F* | 5'-TGTGTGCTCGCGCTCCTATGACACG-3' |
| *OsDT2-Cas9-1-R* | 5'-AAACCGTGTCATAGGAGCGCGAGCA-3' |
| *OsDT2-Cas9-2-F* | 5'-TGTGTGTGATCGTAACCACAAGCAC-3' |
| *OsDT2-Cas9-2-R* | 5'-AAACGTGCTTGTGGTTACGATCACA-3' |
| *OsNF-YC4-Cas9-F* | 5'-TGTGTGCCGGCTGAGGTTGTGATGA-3' |
| *OsNF-YC4-Cas9-R* | 5'-AAACTCATCACAACCTCAGCCGGCA-3' |
| *AtDT2-Cas9-F* | 5'-GATTGGGTCTTTTGGGACTGGATCA-3' |
| *AtDT2-Cas9-R* | 5'-AAACTGATCCAGTCCCAAAAGACCC-3' |
| *gOsDT2-F* | 5'-CGGTATCGATAAGCTTTTTAAATTTAAATTATCAT-3' |
| *gOsDT2-R* | 5'-ATTCGATATCAAGCTTTTGTGAAACATCAGAAT-3' |
| *gOsNF-YC4-F* | 5'-CGGTATCGATAAGCTTTGTTTATATCTGTGCGTGTG-3' |
| *gOsNF-YC4-R* | 5'-ATTCGATATCAAGCTTATCACTTTGCTGCTCT-3' |
| *gAtDT2-F* | 5'-CGGTATCGATAAGCTTTTTATAATGATGGTTGTGTCC-3' |
| *gAtDT2-R* | 5'-ATTCGATATCAAGCTTCTGATGAATTTGTTCAGATTG-3' |
| *gAtABF3-F* | 5'-CGGTATCGATAAGCTTCCTTCTAGGATTTTAGCCTAG-3' |
| *gAtABF3-R* | 5'-ATTCGATATCAAGCTTCCAGGGACCCGTCAATGTCCT-3' |
| *OsDT2-OE-F* | 5'-CGGTATCGATAAGCTTATGATCAAAGAATCTTC-3' |
| *OsDT2-OE-R* | 5'-ATTCGATATCAAGCTTTTGTGAAACATCAGAAT-3' |
| *OsNF-YC4-OE-F* | 5'-CGGTATCGATAAGCTTATGGAGCCATCATCAC-3' |
| *OsNF-YC4-OE-R* | 5'-ATTCGATATCAAGCTTATCACTTTGCTGCTCT-3' |
|  |  |
| Primers for mutant genotyping | |
| *osdt2-Cas9-1-geno-F* | 5'-ATGATCGTAACCACAAGCACCG-3' |
| *osdt2-Cas9-1-geno-R* | 5'-GCATTCCATTAGGCATTCCAGG-3' |
| *osdt2-Cas9-2-geno-F* | 5'-AGTTTGCTGTGGGCTTGG-3' |
| *osdt2-Cas9-2-geno-R* | 5'-GAGGTGCCCGACTATGAG-3' |
| *osnf-yc4-Cas9-geno-F* | 5'-TTGTAACATGGGAGGCTAGGACT-3' |
| *osnf-yc4-Cas9-geno-R* | 5'-TGCTGGAGTTGACGCTGTT-3' |
| *atdt2-Cas9-geno-F* | 5'-CCACCTGATTGTATGAGTGACCC-3' |
| *atdt2-Cas9-geno-R* | 5'-ATCCAAGCCACCATTAGGAACTC-3' |
|  |  |
| Primers for ChIP assays |  |
| *OsLEA3-1-F* | 5'-ATTCTTTTGTCGGCCACCCTCCGC-3' |
| *OsLEA3-1-R* | 5'-GAGCTCAGTTTAGTTCTTGAGATTTC-3' |
| *OsLEA3-2-F* | 5'-GAGGGAGCACTACGGCGCACCGAC-3' |
| *OsLEA3-2-R* | 5'-TGGAGGCCCTTTCGTGCTGCTAGCC-3' |
| *OsLEA3-3-F* | 5'-GATCGACTTGAGTTAATCGGCAAG-3' |
| *OsLEA3-3-R* | 5'-GTATCTGCTCATCTTTTCGAGATCG-3' |
| *OsLEA3-4-F* | 5'-TGTCCCCTTAACATTGTTTACTCCC-3' |
| *OsLEA3-4-R* | 5'-ATCGATCGGGTGTCGTTGCCATGTG-3' |
| *OsLEA3-5-F* | 5'-ACCGATCGATCGACGTTTCGATCGC-3' |
| *OsLEA3-5-R* | 5'-CGGCTGAGAGCTCAGGCACGTGCCG-3' |
| *OsLEA3-6-F* | 5'-AGGATGTCTCGATGCCAACCCTTAT-3' |
| *OsLEA3-6-R* | 5'-ATTAACCTCGAAATTCACGCGCG-3' |
| *OsLEA3-P1-F* | 5'-ACATTGTTTACTCCCTTTGCCGCC-3' |
| *OsLEA3-P1-R* | 5'-GGCTGTTTGGCGAGTACAAGTTGG-3' |
| *OsLEA3-P2-F* | 5'-ACCGATCGATCGACGTTTCGATCGC-3' |
| *OsLEA3-P2-R* | 5'-CGGCTGAGAGCTCAGGCACGTGCCG-3' |
| *OsLEA3-P3-F* | 5'-ATGAGAGGGCTTCGCGAAGGTAC-3' |
| *OsLEA3-P3-R* | 5'-ATGGGATTGGAATGGAACAGTAT-3' |
| *OsLEA14-P1-F* | 5'-TAGTCACCATCTAAGCTACCGGGC-3' |
| *OsLEA14-P1-R* | 5'-CAGTCTTGTGTAATTTTCTTCCGCG-3' |
| *OsLEA14-P2-F* | 5'-TCTCCCGACAGTCATCCATGAGCC-3' |
| *OsLEA14-P2-R* | 5'-GGACAGTTGGAGCGTCTCGTGGAA-3' |
| *OsLEA14-P3-F* | 5'-AAAACACTGAAAAACTGATGACGC-3' |
| *OsLEA14-P3-R* | 5'-GTCGTCGCTGTACTGTGTTAGGAG-3' |
| *OsRab16A-P1-F* | 5'-AACACAATGACACCGTCGAAGAAAGC-3' |
| *OsRab16A-P1-R* | 5'-CGACCACGCTAGTGACCATGAAGC-3' |
| *OsRab16A-P2-F* | 5'-CAATCTTGGTTAAGGGTTGGAAC-3' |
| *OsRab16A-P2-R* | 5'-CACTTGGGGACTGCTATGTATCA-3' |
| *OsRab16A-P3-F* | 5'-GACACCGTACGTGGCGCCACCG-3' |
| *OsRab16A-P3-R* | 5'-GAGACAGGGAGACGTGTGCGGG-3' |
| *OsABA8ox1-P1-F* | 5'-GCGATGAGGATACGTTGGATTA-3' |
| *OsABA8ox1-P1-R* | 5'-TCTGCCATGGTTTCTGGACACG-3' |
| *OsABA8ox1-P2-F* | 5'-TAGGAGCAGGAGCAGGAGCAGG-3' |
| *OsABA8ox1-P2-R* | 5'-CACCTCTCCCTTTCCGCCCTGT-3' |
| *OsABA8ox1-P3-F* | 5'-GCACGCCTTTTTCCCTTTATTTCC-3' |
| *OsABA8ox1-P3-R* | 5'-TTTCTCCTCCGTTTCGCTCTCCTT-3' |
| *OsRab16C-P1-F* | 5'-GCTGCTACTGCTCCCTGTAGACCTGC-3' |
| *OsRab16C-P1-R* | 5'-TCCTCGGAGTTGAGTCCCCCCTAC-3' |
| *OsRab16C-P2-F* | 5'-TTACAAAAGGTGAACTGTGGGAGC-3' |
| *OsRab16C-P2-R* | 5'-AAACCGAAGTGTAGGTCAGGAAGG-3' |
| *OsRab16C-P3-F* | 5'-CAACCTCCAAGATCAGTATAAATGGC-3' |
| *OsRab16C-P3-R* | 5'-CTTCTCAAACACGCGTCTGATCTC-3' |
| *OsRab16B-P1-F* | 5'-GTGTGCCAAATGACGGCTTAAGAG-3' |
| *OsRab16B-P1-R* | 5'-ATCCGATCAAACATCCGATGTGATAG-3' |
| *OsRab16B-P2-F* | 5'-TCATACCTCAAAACACATCCTTGTCG-3' |
| *OsRab16B-P2-R* | 5'-ATGCATACCATCATATCCATGCAGAT-3' |
| *OsRab16B-P3-F* | 5'-ACAAAGTGTCCAGAAGAACCCAACGG-3' |
| *OsRab16B-P3-R* | 5'-ATTTATACCGCGGCGAGACTGTGG-3' |
| *Os05g12040-P1-F* | 5'-TGTCAAAATAATCACATCTATTCCAG-3' |
| *Os05g12040-P1-R* | 5'-GAACTTTCACTCAACATGTGTCTAAC-3' |
| *Os05g12040-P2-F* | 5'-GTTCGGATCTACATACCACGTAT-3' |
| *Os05g12040-P2-R* | 5'-GATCTCCACCTTTATAAGTAGTAC-3' |
| *Os05g12040-P3-F* | 5'-AGGACGTTGGATCTCAAGCAAG-3' |
| *Os05g12040-P3-R* | 5'-CGCACACTTATACGTCTAATGTG-3' |
| *Os03g55320-P1-F* | 5'-CATCCACCGTAGCAAGTTTTTTCTC-3' |
| *Os03g55320-P1-R* | 5'-AGTTCACGACCAAGATCTGCAAGTAG-3' |
| *Os03g55320-P2-F* | 5'-CTTTTGCCTTTCTGGAAGCATCGG-3' |
| *Os03g55320-P2-R* | 5'-TGCTCCTCCACTAGCCTTAGATTCCG-3' |
| *Os03g55320-P3-F* | 5'-ACTTCCTTATCGTGCTACTTGTGC-3' |
| *Os03g55320-P3-R* | 5'-TTGTTGGTTTCAGACAGGTGTAGTT-3' |
| *Os03g41390-P1-F* | 5'-TAATTAGGCTCAAAAGATTCGGCT-3' |
| *Os03g41390-P1-R* | 5'-ATACCCACATATGAATTTACAGCGC-3' |
| *Os03g41390-P2-F* | 5'-CAAATACCAATTGAGGTGGGACTAGC-3' |
| *Os03g41390-P2-R* | 5'-ATGCAACTTAGGATTTGATTGGGTGT-3' |
| *Os03g41390-P3-F* | 5'-GCATGTTCCATTACCGTGCTTCTG-3' |
| *Os03g41390-P3-R* | 5'-GGTTCTTGGGAACTAAGCAAAAGACC-3' |
| *Os03g12820-P1-F* | 5'-AACACACGAACTTCCGTCCGTACT-3' |
| *Os03g12820-P1-R* | 5'-TTGACCATCTGATCATCCTACGGTT-3' |
| *Os03g12820-P2-F* | 5'-GCCAAGAAAAATACGCACAAGAGAG-3' |
| *Os03g12820-P2-R* | 5'-ACTTTGCCCTTTACTTCACCTGGAC-3' |
| *Os03g12820-P3-F* | 5'-GGGCAAACTAGACATTTCTAGACTTC-3' |
| *Os03g12820-P3-R* | 5'-GGAGGTGGGGATGAATATATAGGT-3' |
| *AtLEA3-1-F* | 5'-TCCAAGTTTTTGCTGTCGTATGA-3' |
| *AtLEA3-1-R* | 5'-CCTAAACTAAATTGAGTCGGAAC-3' |
| *AtLEA3-2-F* | 5'-ATGCAATCACAATTATTTAGGAG-3' |
| *AtLEA3-2-R* | 5'-ACTATAGTTTGACCAAAACCAAT-3' |
| *AtLEA3-3-F* | 5'-CCAGATAAGTAAAACAAGTTGTT-3' |
| *AtLEA3-3-R* | 5'-ATAGATGAGAAGGAAGAGAGATG-3' |
| *AtLEA3-4-F* | 5'-CAAGAGAGATAGGACGACCAAGT-3' |
| *AtLEA3-4-R* | 5'-TTAGCGAGAGAACGAGCCATTGT-3' |
| *AtLEA3-5-F* | 5'-ACGCCGTTTTCAGGTAACATCTG-3' |
| *AtLEA3-5-R* | 5'-AAACCCTCGTCTGAAGAGAATCC-3' |
